# P2X7 receptor inhibition alleviates mania-like behavior independently of interleukin-1β

**DOI:** 10.1016/j.isci.2024.109284

**Published:** 2024-02-20

**Authors:** Flóra Gölöncsér, Mária Baranyi, Pál Tod, Fruzsina Maácz, Beáta Sperlágh

**Affiliations:** 1 Laboratory of Molecular Pharmacology, HUN-REN Institute of Experimental Medicine, 1083 Budapest, Hungary; 2 János Szentágothai School of Neurosciences, Semmelweis University School of Ph.D Studies, 1083 Budapest, Hungary

**Keywords:** Behavioral neuroscience, Biological sciences, Natural sciences, Neuroscience, Pharmacology

## Abstract

Purinergic dysfunctions are associated with mania and depression pathogenesis. P2X7 receptor (P2X7R) mediates the IL-1β maturation via NLRP3 inflammasome activation. We tested in a mouse model of the subchronic amphetamine (AMPH)-induced hyperactivity whether P2X7R inhibition alleviated mania-like behavior through IL-1β.

Treatment with JNJ-47965567, a P2X7R antagonist, abolished AMPH-induced hyperlocomotion in wild-type and IL-1α/β-knockout male mice. The NLRP3 inhibitor MCC950 failed to reduce AMPH-induced locomotion in WT mice, whereas the IL-1 receptor antagonist anakinra slightly increased it. AMPH increased IL-10, TNF-α, and TBARS levels, but did not influence BDNF levels, serotonin, dopamine, and noradrenaline content in brain tissues in either genotypes. JNJ-47965567 and P2rx7-gene deficiency, but not IL-1α/β-gene deficiency, attenuated AMPH-induced [^3^H]dopamine release from striatal slices. In wild-type and IL-1α/β-knockout female mice, JNJ-47965567 was also effective in attenuating AMPH-induced hyperlocomotion.

This study suggests that AMPH-induced hyperactivity is modulated by P2X7Rs, but not through IL-1β.

## Highlights

• P2X7R antagonist reduced AMPH-induced hyperactivity independent of NLRP3 and IL-1αβ
• P2X7R antagonist reduced IL-10 and TNF-α levels in mania activating brain regions
• P2X7R antagonist reduced [^3^H]DA release from the striatum, independent of IL-1αβ
• P2X7KO mice showed reduced social and aggressive contacts in resident-intruder test

## Graphical abstract

**Figure:**
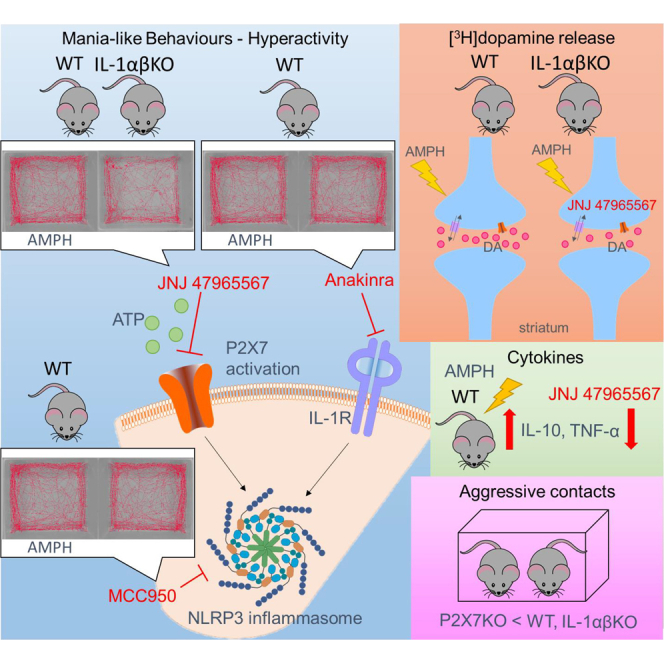

## Introduction

Bipolar mania is the manic phase of bipolar disorder (BD), a pathological period in which patients experience drastic changes in mood and behavior that affect their lives. This period is associated with elevated or irritable moods, increased activity and energy, as well as increased self-esteem, reduced need for sleep, talking more than usual, racing thoughts, and excessive participation in high-risk activities.^1^ Mood disorders, including BD, are thought to result from the interaction of genetic, environmental, psychological, and social factors. Studies have shown that specific brain regions^2^^,^^3^ and aberrant immune signals may be involved in the pathophysiology of BD.^4^^,^^5^^,^^6^ Despite differences within studies, overall serum concentrations of tumor necrosis factor (TNF)-α, soluble TNF-receptor 1, interleukin (IL)-1β, IL-4, soluble IL-2, and IL-6 receptors appear to be consistently higher in manic and depressive phases than in healthy controls.^7^^,^^8^ A meta-analysis of 30 clinical studies showed significantly higher peripheral IL-1β levels in patients with BD,^9^ and IL-1β levels were also increased in the cerebrospinal fluid of patients with BD compared to controls.^10^

Mania is associated with the overactivity of the dopaminergic system. The use of AMPH increases dopamine (DA) efflux, inhibits DA reuptake and reduces DA degradation by MAO,^11^ and increases locomotor activity, reflecting hyperactivity and psychomotor symptoms in manic episodes of BD. Acute or subchronic/chronic AMPH-induced rodent models of mania show predictive, construct, and face validity.^12^^,^^13^ These models have been used repeatedly as a tool to investigate novel therapeutic targets with potential mood stabilizing properties.^14^ They therefore appear to be reliable and useful in preclinical studies of BD. However, it should not be overlooked that manic episodes are clearly more complex than DA enhancement itself.

The purinergic system plays a role in the regulation of pathophysiological changes in mood and activity. Patients with mania have elevated uric acid levels, which may indicate the dysfunction of the purinergic system.^15^^,^^16^^,^^17^ Purinergic signaling is mediated by specific purinergic membrane receptors activated by extracellularly released nucleosides (P1 adenosine receptors) and nucleotides (P2Y and P2X receptors). The homomeric P2X7R is a unique member of the ATP-gated ionotropic P2X receptor family with low ATP affinity. It is also slow to desensitize, can induce plasma membrane permeabilization for large molecules, and is a major driver of inflammation.^18^ Previous studies have shown that the inhibition of P2X7Rs activated by extracellular ATP has both antidepressant and anti-manic effects in animal models.^19^^,^^20^^,^^21^^,^^22^^,^^23^^,^^24^^,^^25^ However, the mechanisms underlying these two kinds of effects of P2X7 inhibition are not necessarily the same. It is important to note that in a human proof-of-concept study, the selective P2X7R antagonist JNJ-54175446 showed mood-altering effects during dexamphetamine challenge^26^ and is currently being investigated in a Phase II clinical trial for the treatment of resistant depression (ClinicalTrials.gov Identifier: NCT04116606).^27^ However, previous data on the signaling mechanism, whereby the inhibition of P2X7R leads to the attenuation of AMPH-induced hyperactivity are contradictory.^19^^,^^20^^,^^24^ Previously, we and others have shown that the absence of the P2rx7 gene in mice attenuates AMPH-induced hyperlocomotion and DA release in the striatum.^19^^,^^21^ Furthermore, the antimanic effect of P2rx7 gene deficiency was not transferable to recipient mice by bone marrow transplantation, suggesting that P2X7R expressed by non-hematopoietic cells may be responsible for this effect.^21^ Other studies have confirmed the involvement of the dopaminergic system and astrogliosis, or suggested a role for the inflammatory response system in the mechanism of action P2X7 inhibition.^19^^,^^24^ It is well known that P2X7R activation regulates the expression of proinflammatory cytokines such as IL-6, TNF-α, and IL-1β, and the latter may be associated with the antimanic effect of P2X7R inhibition.^17^^,^^28^^,^^29^ In peripheral and central immune cells, ATP-activated P2X7R promotes the NLRP3 (NOD-like receptor protein 3) inflammasome-mediated activation of caspase-1 and leads to IL-1β secretion^30^^,^^31^ following priming stimulus. Therefore, the possibility arises that IL-1β is also involved in ameliorating the effect of P2X7R inhibition on manic behavior.

Monoaminergic psychostimulant drugs, such as AMPH, induces long-lasting changes in the reward system. AMPH increases the availability of DA through increased release, the depletion of vesicular contents, membrane transporter reversal, and decreased DA metabolism by inhibition monoamine oxidase activity.^32^ Previous studies have also demonstrated the involvement of P2X7R in the regulation of monoaminergic neurotransmission. Hippocampal serotonin (5-HT) and noradrenaline (NA) release is modulated by the endogenous activation of P2X7R.^33^^,^^34^ In the brain, the activated central inflammatory response also influences neurotransmitter systems via metabolic and functioning of presynaptic pumps.^35^ Proinflammatory cytokines may affect several aspects of DA neurotransmission, leading to reduced synthesis, impaired packaging or release, or increased uptake,^35^^,^^36^ and, conversely, dopamine receptors may also regulate the release of inflammatory mediators and subsequent pathological processes by interacting with inflammasomes, inflammatory pathways.^37^

In the present study, we asked whether IL-1β is involved in the mechanism of P2X7R inhibition that attenuates AMPH-induced hyperactivity. Previous literature data suggest, albeit inconclusively, that IL-1β may play an important role in the AMHP-induced animal model of mania and that P2X7R antagonists may exert their effects by blocking this signaling pathway. To find out, in our experiments, we used non-inducible IL-1αβKO mice, P2X7R, IL-1 receptor antagonist, and NLRP3 inflammasome inhibitor. In addition, we examined the social and aggressive behavior of IL-1αβKO, P2X7KO and WT mice in an environmental mania model. We also investigated the modulatory effects of subchronic AMPH treatment and P2X7R inhibition on cytokines, monoamines, TBARS, and BDNF in brain areas relevant in manic behavior.

## Results

### Phenotyping of the interleukin-1αβ knockout mice

**Figure 1:**
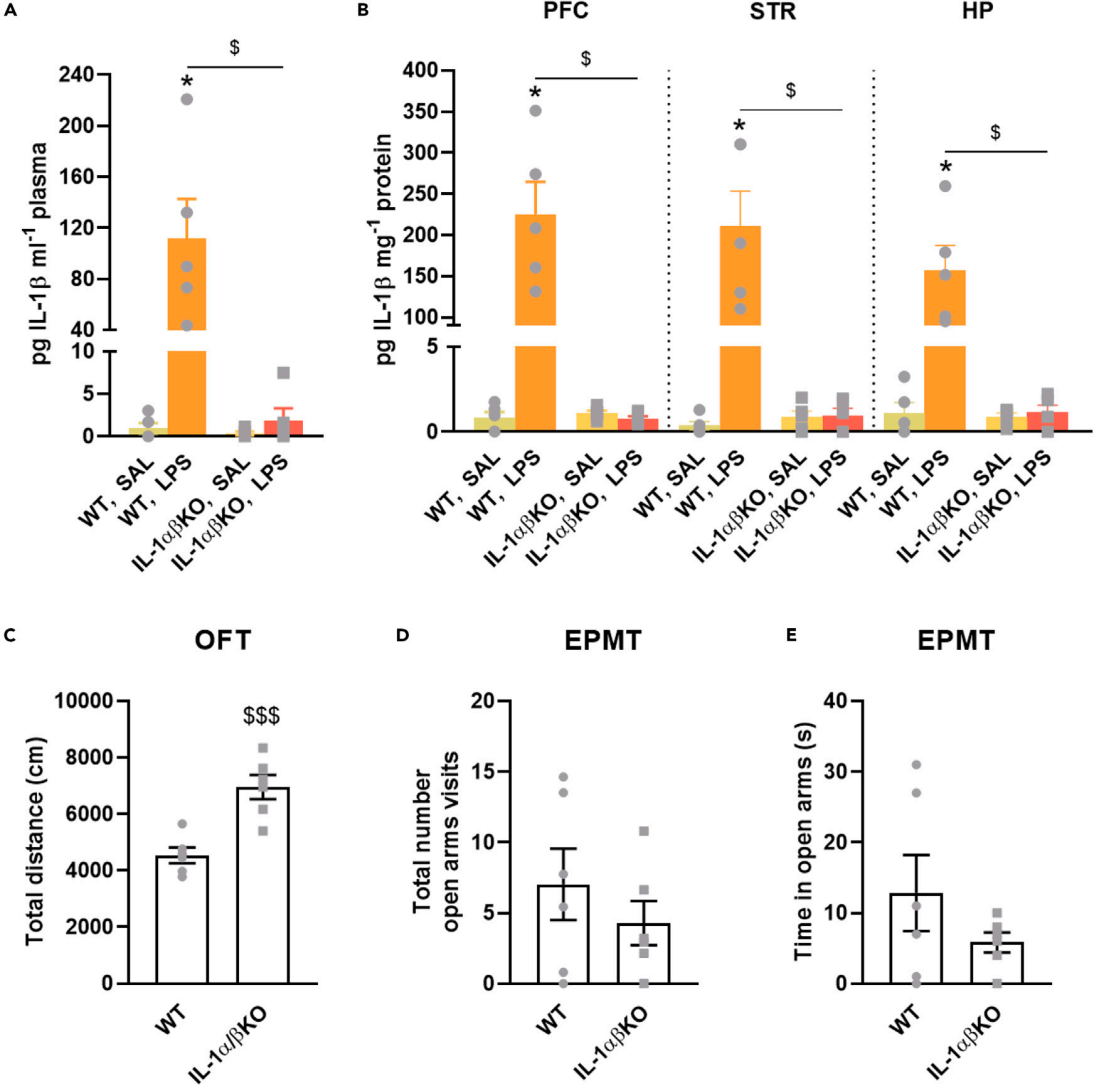
IL-1β levels in plasma and brain areas 6 h after LPS treatment and behavioral phenotypic characterization of IL-1αβKO mice (A and B) Expression of IL-1β levels was measured by ELISA in WT and IL-1αβKO mice. Values are presented as means ± SEM of n = 5 mice per group. (C) Total distance traveled in the open field test. Values are presented as means ± SEM of n = 6 mice per group. (D and E) Total number of open arms visits and cumulative duration in open arms in the elevated plus maze test. Mice were placed in the center of the arena and left free for 5 min. Values are presented as means ± SEM of n = 6 mice per group. Values are presented as means ± SEM of n = 6 mice per group. Kruskal-Wallis test with multiple comparisons (A and B), Student’s *t* test (C–E): ∗p < 0.05 compared SAL treated group in a same genotype; ^$^p < 0.05, ^$$$^p < 0.001 compared to the same treatment (or naive) group with WT (or naive). EPMT, elevated plus maze test, FST, HP, hippocampus, IL, interleukin; IL-1αβKO, interleukin-1α/β knockout mice, LPS, lipopolysaccharides, OFT, open field test, PFC, prefrontal cortex, SAL, saline, STR, striatum, WT, wild-type mice. Additional results from the behavioral tests can be found in Figure S3 and S4.

To validate the gene deficiency of IL-1α/β knockout (IL-1αβKO) mice, we examined plasma and brain IL-1β levels in WT and IL-1αβKO mice under basal conditions and after endotoxin challenge. While basal plasma and brain IL-1β levels were similar in the low pg ml^−1^ range in WT and IL-1αβKO mice, a single injection of 1 mg kg^−1^ lipopolysaccharide (LPS) caused a significant but moderate increase in IL-1β levels in the plasma of WT mice, but did not affect brain IL-1β levels (See Figure S2). Therefore, we also tested a higher dose of LPS (20 mg kg^−1^), which caused a robust induction of IL-1β levels in plasma and brain regions of WT mice 6 h after injection, but not in IL-1αβKO mice (Figures 1A and 1B). In addition, naive IL-1αβKO mice exhibited a hyperactive behavioral phenotype characterized by hyperlocomotion in the open field arena and elevated plus maze test, but no change in anxiety/novel exploration in the elevated plus maze test and the light-dark box test (See Figures 1C–1E, S3, and S4).

### The AMPH-induced hyperlocomotion did not change in interleukin-1αβ knockout male mice, and JNJ-47965567 (JNJ) inhibited in both genotypes

**Figure 2:**
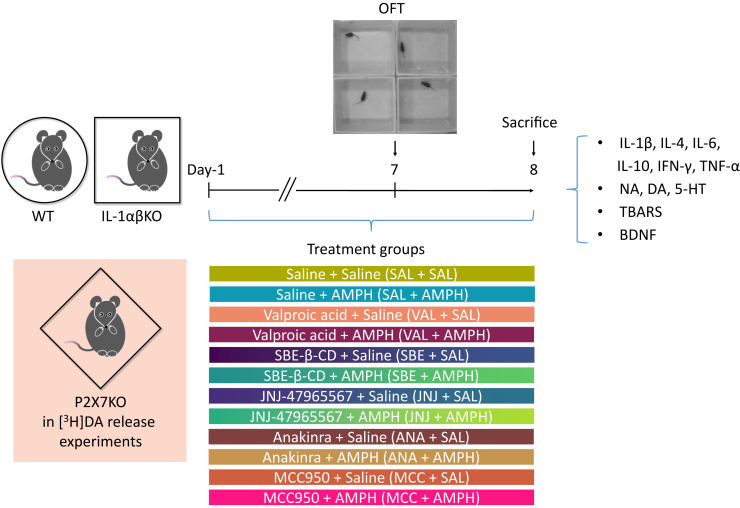
Schematic diagram of experimental study design showing treatment groups, examined factors and chronogram This *in vivo* experimental system replicates aspects of the human manic behavior in mice, which is used to investigate the molecular basis of mania. Hereafter, the circle indicates WT mice, the square indicates IL-1αβKO mice, and the rhombus indicates P2X7KO mice in the experiments shown in the figures. In addition, the color of the treatment groups is consistently the same. AMPH, d-amphetamine, BDNF, brain-derived neurotrophic factor, DA, dopamine, IFN, interferon, IL, interleukin; IL-1αβKO, interleukin-1α/β knockout mice, NA, noradrenaline, OFT, open field test, P2X7KO, P2X7 receptor knockout mice, SBE, beta cyclodextrin sulfobutyl ether, TBARS, thiobarbituric acid reactive substances, TNF, tumor necrosis factor, VAL, valproic acid sodium salt, WT, wild-type mice, 5-HT, serotonin.

In an AMPH-induced mania model, we tested whether the subchronic inhibition of P2X7R could modify AMPH-induced behavior in mice by administering the P2X7R-selective antagonist JNJ 30 min before AMPH administration for 7 consecutive days. Figure 2 shows a schematic diagram of the experimental study design, treatment groups, examined factors, and chronogram.

**Figure 3:**
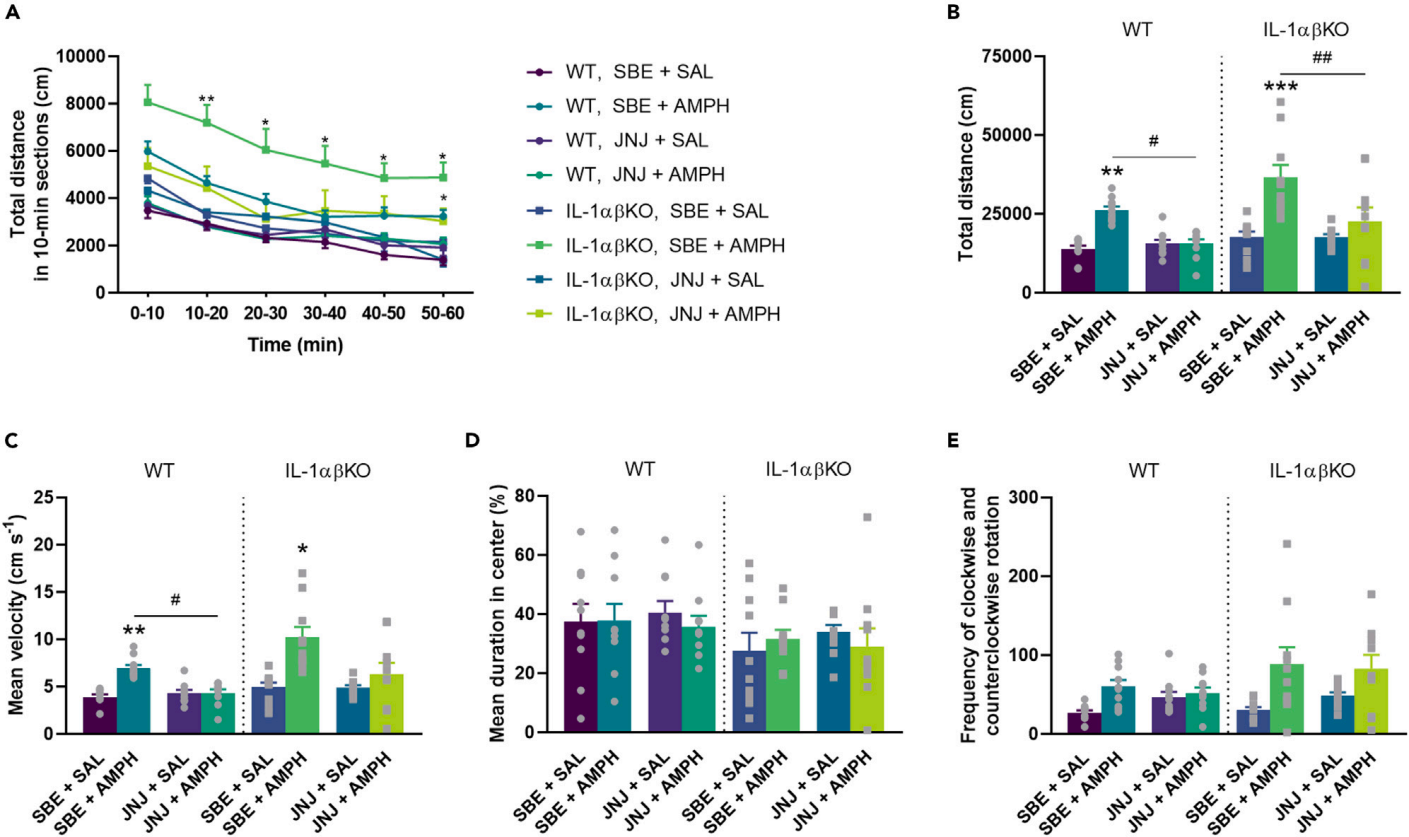
JNJ significantly decreased AMPH-induced locomotion in WT and IL-1αβKO mice Mice were treated with JNJ/SBE and 30 min later with AMPH/SAL once a day for 7 consecutive days. Immediately after the last AMPH injection, mice were subjected to the open field apparatus. (A) Distance traveled by time (time × treatment: F[15,360] = 3.404, p = 0.00002). (B) Total distance traveled. AMPH increased locomotion significantly in both genotypes (●, ▪) which was decreased by JNJ (●, ▪). (C) Velocity. AMPH increased velocity significantly in both genotypes (●, ▪) which was decreased by JNJ (●, ▪). (D) Cumulative duration in central zone. (E) Circling behavior of mice. Values are presented as means ± SEM of n = 10 mice per group. Repeated Measures ANOVA (A), after square root transformation (B),two-way ANOVA (D) followed by Tukey’s multiple comparison *post hoc* test, and Kruskal-Wallis test with multiple comparisons (C and E): ∗p < 0.05, ∗∗p < 0.01, ∗∗∗p < 0.001 compared to SBE + SAL treated group in a same genotype; ^#^p < 0.05, ^##^p < 0.01 compared to the SBE + AMPH treated group in a same genotype. AMPH, d-amphetamine, IL-1αβKO, interleukin-1α/β knockout mice, JNJ, JNJ-47965567, SAL, saline, SBE, beta cyclodextrin sulfobutyl ether, WT, wild-type mice.

After repeated SBE + AMPH (●, ▪), we observed a significant increase in the distance traveled and mean velocity of mice in both genotypes compared to the SBE + SAL (●, ▪) groups (Figures 3A–3C). However, there was no significant difference in AMPH-induced locomotor activity between WT and IL-1αβKO mice (●, ▪, Figures 3B and 3C). While the administration of JNJ (●, ▪) significantly reduced AMPH-induced motility in both genotypes (●, ▪; Figures 3A–3C), the basal activity of mice was not affected compared to SBE + SAL (●, ▪). In addition, AMPH or JNJ treatment had no effect on the time spent in the central zone of mice (Figure 3D) or circling behavior of mice (Figure 3E). These results indicate that the selective inhibition of P2X7R in both genotypes reduces AMPH-induced behavioral activity. Repeated JNJ did not cause changes in the body weight of WT and IL-1αβKO mice (See Figure S5).

### The effect of P2X7R inhibition on amphetamine-induced changes in cytokine levels in mania-related areas of the central nervous system

**Figure 4:**
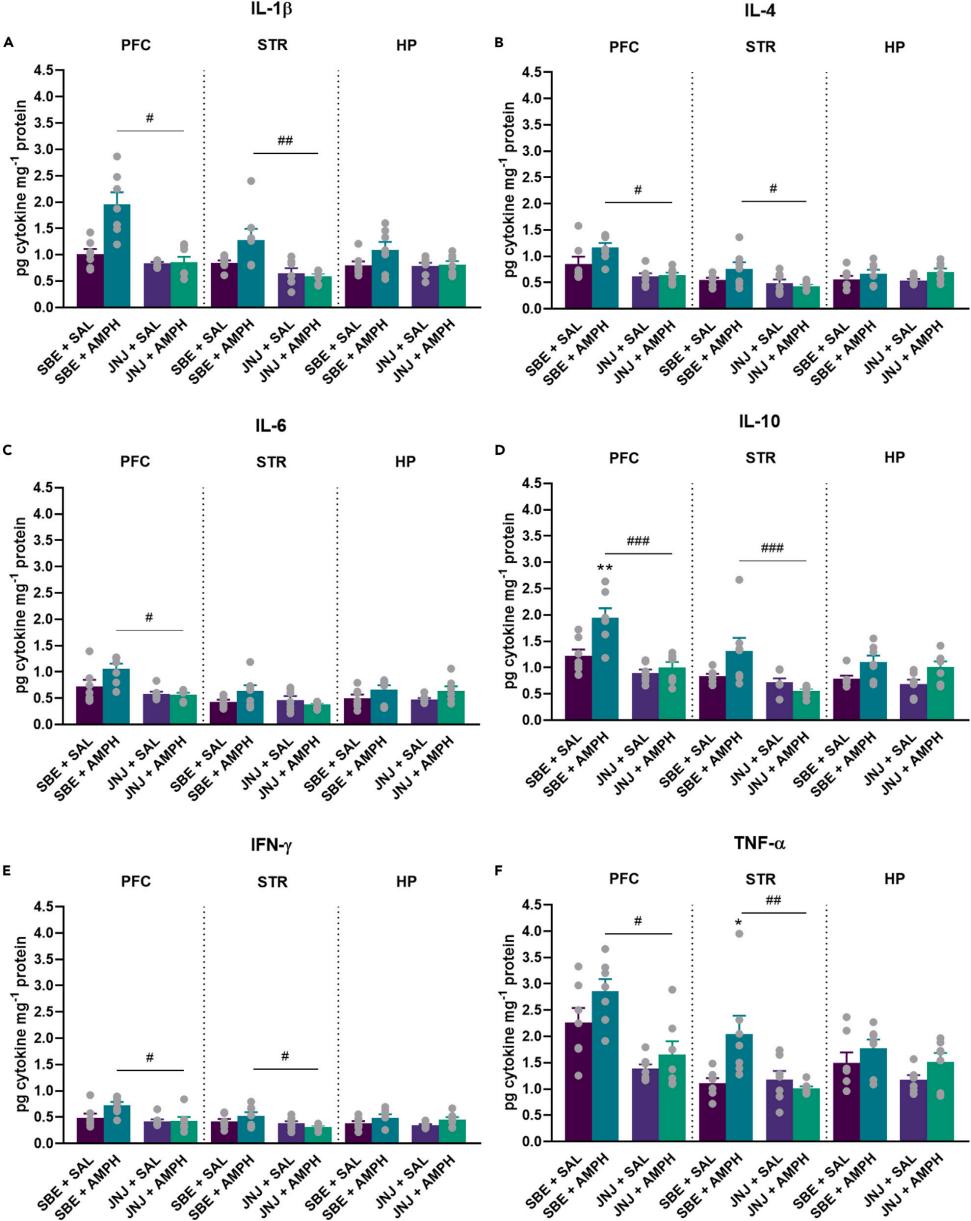
Inflammatory cytokine levels in the PFC, STR and HP 1 h after last JNJ/SBE + AMPH/SAL injections Expression of pro- and anti-inflammatory cytokine levels were measured by CBA FACS analysis in WT mice. (A) IL-1β. (B) IL-4. (C) IL-6. (D) IL-10. (E) IFN-γ. (F) TNF-α. Values are presented as means ± SEM of n = 7 mice per group. We compared the effect of treatments on cytokine levels per brain area. One-way ANOVA followed by Tukey’s multiple comparison *post hoc* test (with or without square root transformation) or Kruskal-Wallis test with multiple comparisons was done: ∗p < 0.05, ∗∗p < 0.01 compared to the SBE + SAL treated group, ^#^p < 0.05, ^##^p < 0.01, ^###^p < 0.001 compared to the SBE + AMPH treated group in a same genotype. AMPH, d-amphetamine, HP, hippocampus, IFN, interferon; IL, interleukin; JNJ, JNJ-47965567, PFC, prefrontal cortex, SAL, saline, SBE, beta cyclodextrin sulfobutyl ether, STR, striatum, TNF, tumor necrosis factor, WT, wild-type mice.

**Figure 5:**
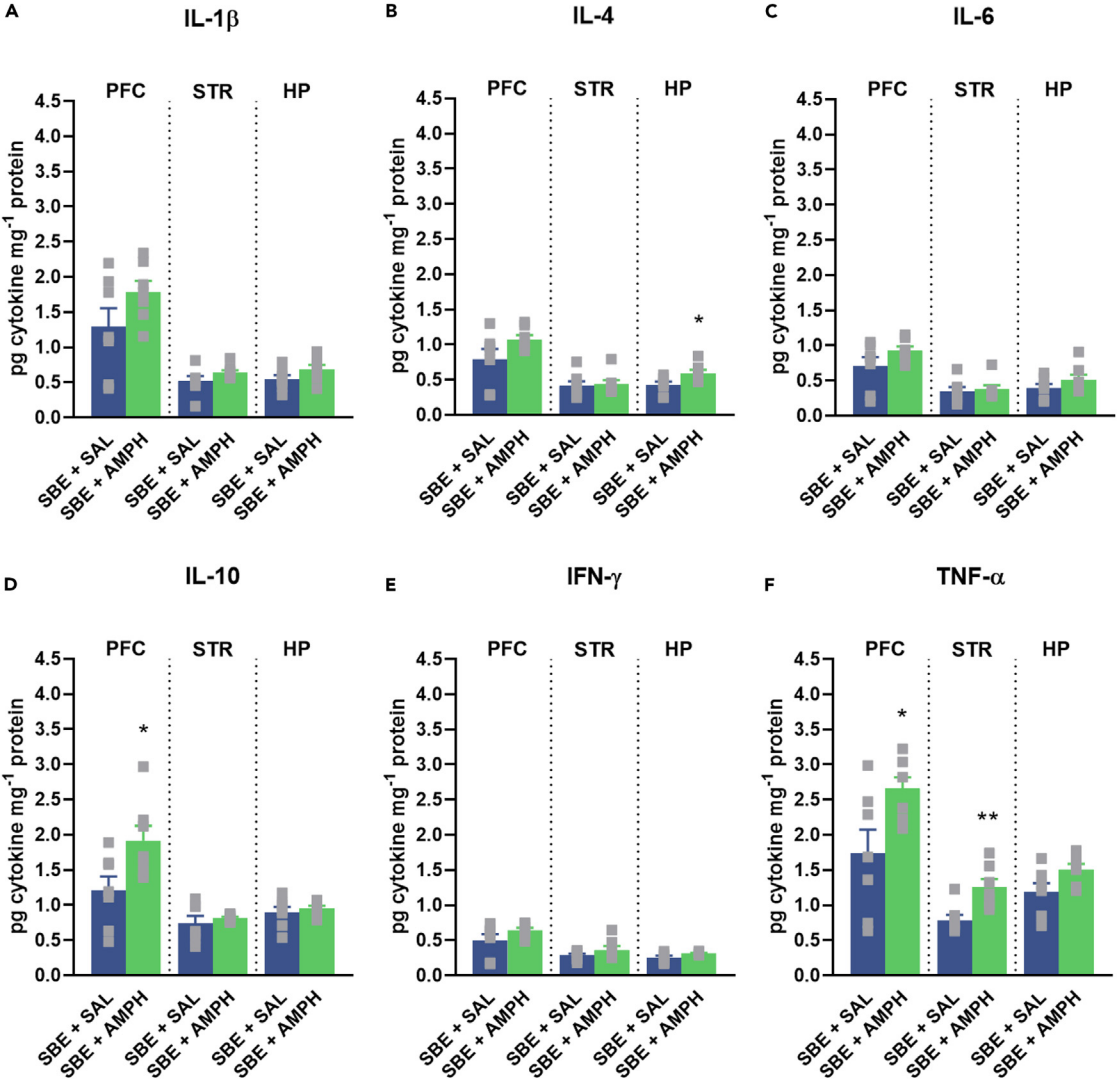
Inflammatory cytokine levels in the PFC, STR and HP 1 h after last SBE + AMPH/SAL injections in IL-1αβKO mice Expression of pro- and anti-inflammatory cytokine levels were measured by CBA analysis in IL-1αβKO mice. (A) IL-1β. (B) IL-4. (C) IL-6. (D) IL-10. (E) IFN-γ. (F) TNF-α. Values are presented as means ± SEM of n = 7 mice per group. We compared the effect of AMPH treatment on cytokine levels in different brain areas. Mann-Whitney U Test or Student’s *t* test were done (with or without square root transformation) were done: ∗p < 0.05, ∗∗p < 0.01 compared SBE + SAL treated group. AMPH, d-amphetamine, HP, hippocampus, IFN, interferon; IL, interleukin; IL-1αβKO, interleukin-1α/β knockout mice, PFC, prefrontal cortex, SAL, saline, SBE, beta cyclodextrin sulfobutyl ether, STR, striatum, TNF, tumor necrosis factor.

To investigate whether pro- and anti-inflammatory cytokines are upregulated in mania-related CNS areas following AMPH treatment, we used multiplex bead array analyses to analyze the production of IL-1β, IL-4, IL-6, IL-10, interferon (IFN)-γ and TNF-α in the prefrontal cortex (PFC), striatum (STR) and hippocampus (HP) (see Figures 4 and 5). Repeated AMPH increased the levels of IL-10 in the PFC and TNF-α in the STR in WT mice (●, Figures 4D and 4F), which were reduced by JNJ. While JNJ decreased the levels of all cytokines tested, mainly in the PFC and STR compared to the SBE + AMPH group.

### Amphetamine-induced changes in cytokine levels in mania-related areas of the central nervous system in interleukin-1αβ knockout mice

Our result showed that, similar to LPS treatment and WT mice, repeated AMPH did not upregulate IL-1β in IL-1αβKO mice in any brain area (Figure 5). However, similar to WT mice, AMPH treatment upregulated IL-10 levels in the PFC and TNF-α levels in the PFC and STR (Figures 5D and 5F), as well as IL-4 levels in the HP.

### Anakinra slightly enhances amphetamine-induced locomotion in wild type mice

**Figure 6:**
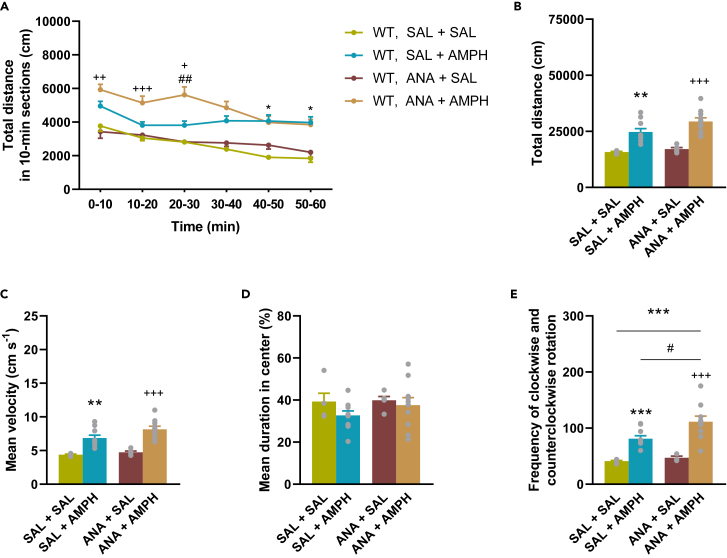
The effect of ANA on AMPH-induced locomotion in WT mice Mice were treated with SAL or ANA and 30 min later with AMPH once a day for 7 consecutive days. Immediately after the last AMPH injection, mice were subjected to the open field apparatus. (A) Distance traveled by time (time × treatment: F[15,80] = 1.729, p = 0.0614). ANA slightly increased AMPH-induced locomotion in WT mice in 20–30 min section (●). (B) Total distance traveled. (C) Velocity. (D) Cumulative duration in central zone. (E) Circling behavior of mice. ANA significantly increased AMPH-induced circling behavior in WT mice (●). Values are presented as means ± SEM of n = 5–10 mice per group. Repeated Measures ANOVA (A) and One-way ANOVA (B, C, D, E (square root transformation)) followed by Tukey’s multiple comparison *post hoc* test: ∗p < 0.05, ∗∗p < 0.01, ∗∗∗p < 0.001 compared to SAL + SAL treated group; ^#^p < 0.05, ^##^p < 0.01 compared to SAL + AMPH treated group; ^+^p < 0.05, ^++^p < 0.01, ^+++^p < 0.001 compared to ANA + SAL treated group. AMPH, d-amphetamine, ANA, anakinra, SAL, saline, WT, wild-type mice.

To investigate whether blockade of the IL-1 receptor plays a role in our AMPH-induced manic model, we treated WT mice with anakinra (ANA). ANA had no effect on basal and AMPH-induced locomotion in WT mice in the open field test (Figure 6B), but slightly enhanced AMPH-induced motility during the 20–30 min section compared to the SAL + AMPH group (Figure 6A). In addition, ANA had no effect on the time spent in the central zone of mice (Figure 6D), but increased the AMPH-induced circling behavior of mice (Figure 6E). These results indicate that the selective inhibition of the IL-1 receptor modestly modulates AMPH-induced behavioral activity. Repeated ANA did not cause any changes in the body weight of WT mice (See Figure S5).

### MCC950 failed to reduce amphetamine-induced locomotion in wild type mice

**Figure 7:**
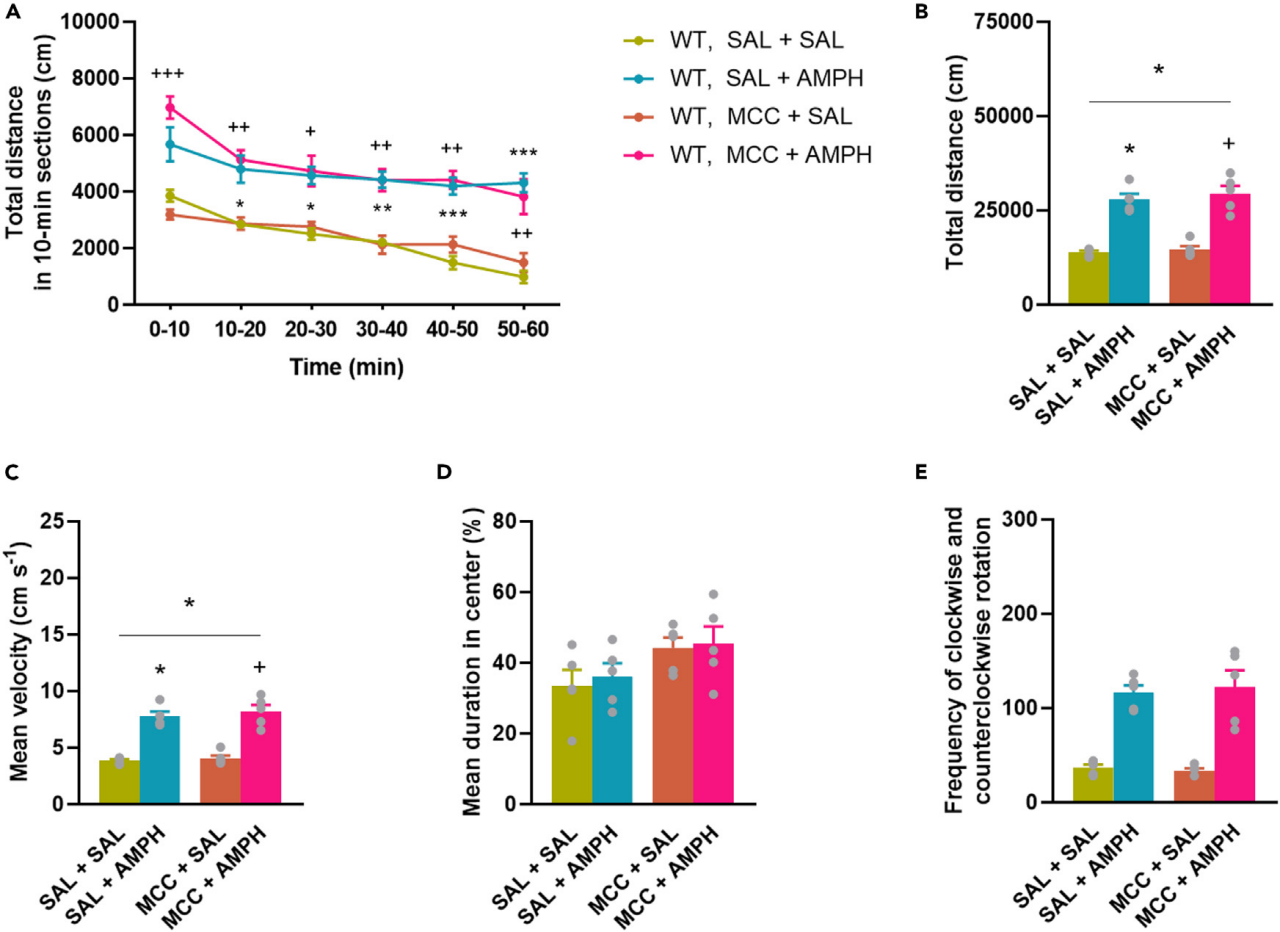
MCC did not affect AMPH-induced locomotion in WT mice Mice were treated with SAL or MCC and 30 min later with AMPH once a day for 7 consecutive days. Immediately after the last AMPH injection, mice were subjected to the open field apparatus. (A) Distance traveled by time. (B) Total distance traveled. (C) Velocity. (D) Cumulative duration in central zone. (E) Circling behavior of mice. Values are presented as means ± SEM of n = 5 mice per group. Repeated Measures ANOVA (A), One-way ANOVA (D, E (after square root transformation)) followed by Tukey’s multiple comparison *post hoc* test and Kruskal-Wallis test with multiple comparisons (B, C): ∗p < 0.05, ∗∗p < 0.01, ∗∗∗p < 0.001 compared to SAL + SAL treated group; ^+^p < 0.05, ^++^p < 0.01, ^+++^p < 0.001 compared to ANA + SAL treated group. AMPH, d-amphetamine, MCC, MCC950, SAL, saline, WT, wild-type mice.

To test whether the NLRP3 inflammasome activation is activated in our AMPH-induced manic model, we treated WT mice with MCC950 (MCC). MCC administered 30 min before AMPH had no effect on the basal and AMPH-induced locomotion of WT mice in the open field test (Figures 7A–7C). In addition, MCC had no effect on the time spent in the central zone of mice (Figure 7D) and the AMPH-induced circling behavior of mice (Figure 7E). These results indicate that the inhibition of the NLRP3 inflammasome does not affect AMPH-induced behavioral activity. Repeated MCC did not cause any changes in the body weight of WT mice (See Figure S5).

### Difference of aggressive behavior in interleukin-1α/β and P2X7R gene-deficient mice

**Figure 8:**
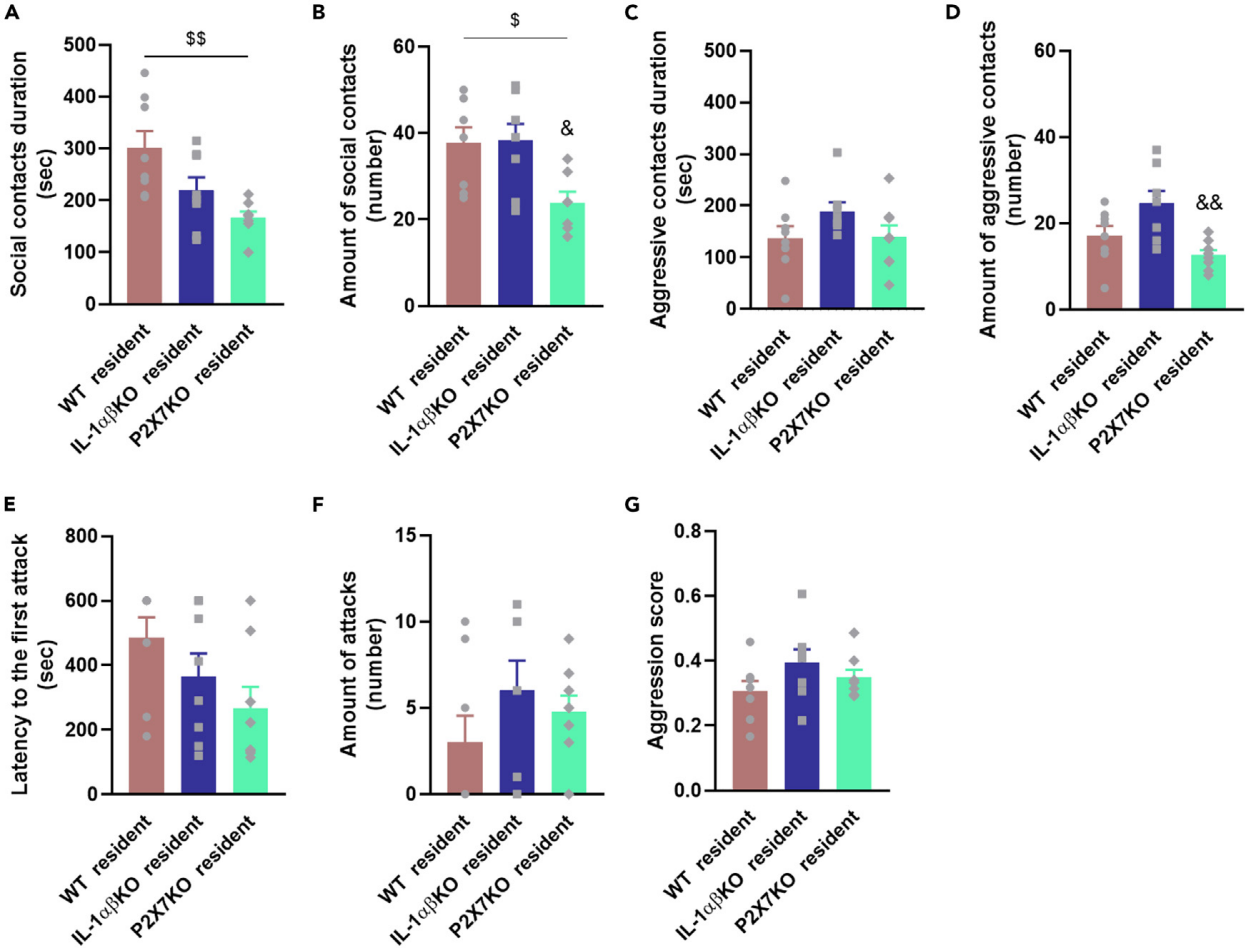
Social and aggressive behavior of mice (A and B) Duration and number of social contacts (sniffing, allogrooming, following) between resident and intruder mice during the test session (duration: genotype: F[2,21] = 7.4790, p = 0.003527; number: F[1,21] = 5.8926, p = 0.009304). (C and D) Duration and number of aggressive contacts (aggressive grooming, upright posture, boxing, chasing, tail rattling, clinch, wrestling, dominant posture, keeping down) of resident mice on an intruder during the session (number: F[2,21] = 7.4509, p = 0.003586). (E, F, and G) Analysis of latency to first attack (E) and number of attacks (F). (G) Aggression score of mice (the ratio of aggressive interactions out of total aggressive + non-aggressive interactions). Values are presented as means ± SEM of n = 8 mice per group. One-way ANOVA (A (square root transformation), B, C, D, G) followed by Tukey’s multiple comparison *post hoc* test and Kruskal-Wallis test with multiple comparisons (E, F): ^$^p < 0.05, ^$$^p < 0.01 compared to WT residents; ^&^p < 0.05 compared IL-1αβKO residents. IL-1αβKO, interleukin-1α/β knockout mice, P2X7KO, P2X7 receptor knockout mice, WT, wild-type mice.

To investigate the aggressive behavior of mice associated with mania-related behavior, mice were subjected to a resident-intruder test (Figure 8). As expected, resident mice showed aggressive behavior toward intruders, but to different degrees. After a month of solitary confinement, P2X7KO mice were less social than their WT counterparts (Figures 8A and 8B) and developed less aggressive contact than IL-1αβKO mice (Figure 8D). There were no differences between mice in the latency and number of aggressive attacks or in their aggression scores (Figures 8E–8G).

### JNJ and P2rx7-gene deficiency, but not interleukin-1α/β-gene deficiency alleviates amphetamine-induced release of [^3^H]DA from striatum

**Figure 9:**
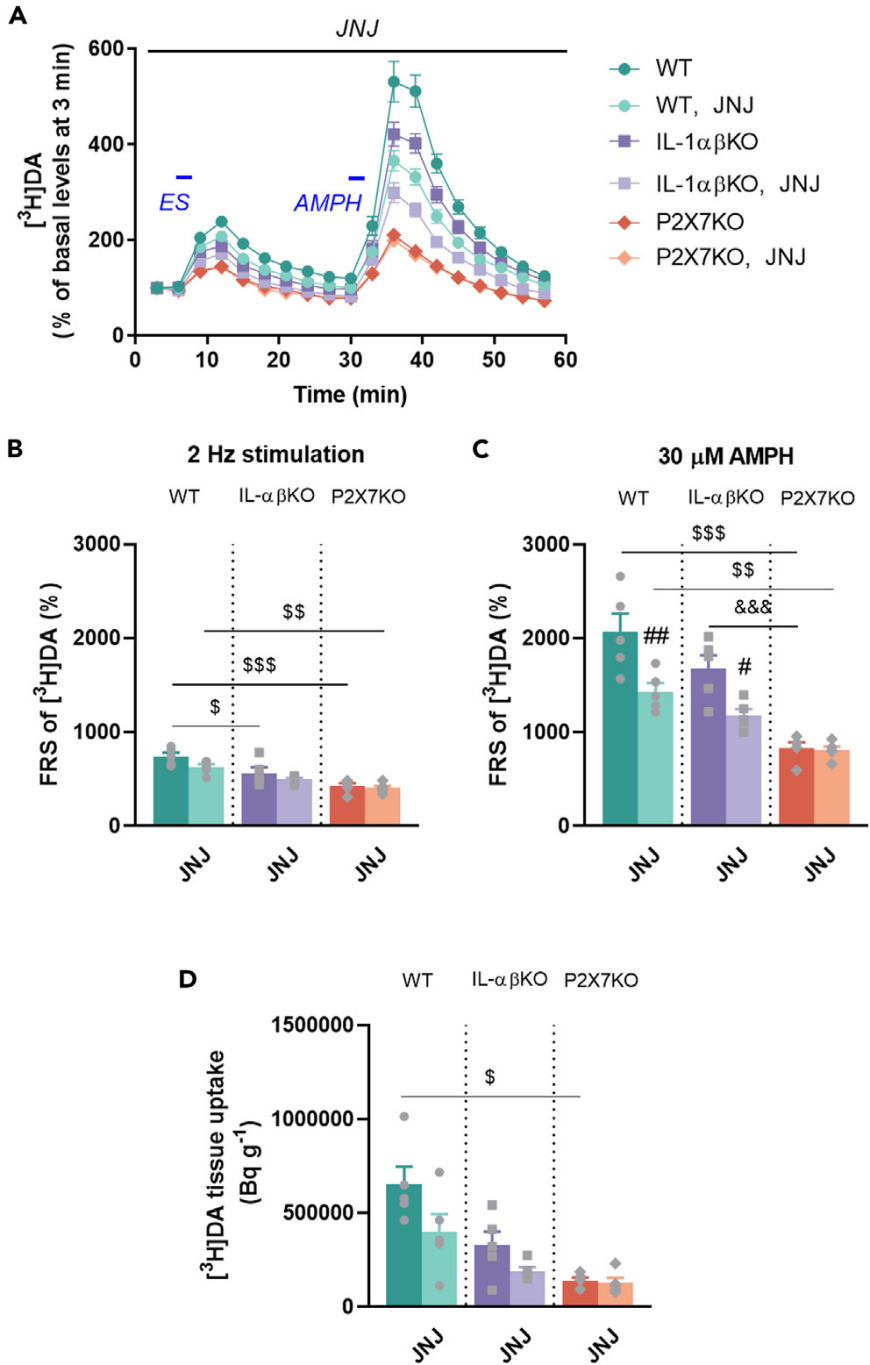
The effect of genetic deletion of IL-1α/β and P2X7R on electrical- and AMPH-evoked [^3^H]DA efflux in striatal slices [^3^H]DA release was measured from superfused coronal striatal slices in response to electrical (40 V, 2 Hz, 1 ms, 2 min) and chemical (30 μM AMPH for 3 min) stimulation. *JNJ* shows the duration of administration to the perfusion solution. (A) [^3^H]DA release in response to electrical (ES) and chemical (AMPH) stimulation. Repeated Measures ANOVA revealed a significant main effect for genotype and pretreatment over time (intercept: F[1,24] = 1645.308, p < 0.0000001; time × genotype × pretreatment: F[34,408] = 3.009, p < 0.0000001). The tritium content in the perfusate samples was expressed as percentage of basal levels and is shown as a function of time. Curves show the means ± SEM of the identical experiments. Blue lines and labels indicate the time of different type of stimulations. (B) Electric field stimulation evoked release of [^3^H]DA (Factorial ANOVA, intercept: F[1,24] = 1295.984, p < 0.0000001. (C) Chemical stimulation evoked release of [^3^H]DA (Factorial ANOVA, intercept: F[1,24] = 816.1452, p < 0.0000001). The tritium content in the perfusate samples was expressed as fractional release (FRS). (D) Tritium uptake of tissue in Bq/g (Kruskal-Wallis test: H(5,N = 30) = 18.00129, p = 0.0029). Values are presented as means ± SEM of n = 5 mice per group. Repeated Measures (A), Factorial ANOVA (B, C) followed by Tukey’s multiple comparison *post hoc* test, and Kruskal-Wallis test with multiple comparisons (D): ^#^p < 0.05, ^##^p < 0.01 compared to non-JNJ-pre-perfused group in same genotype; ^$^p < 0.05, ^$$^p < 0.01, ^$$$^p < 0.001 compared to the same treatment group with WT; ^&&&^p < 0.001 compared to the same treatment group with IL-1αβKO. AMPH, d-amphetamine stimulation, DA, dopamine, ES, electrical stimulation, IL-1αβKO, interleukin-1α/β knockout mice, JNJ, JNJ-47965567, P2X7KO, P2X7 receptor knockout mice, WT, wild-type mice, [^3^H], tritium.

To investigate the effect of AMPH on striatal DA release, we measured the electrical- and AMPH-induced release of [^3^H]DA from acute striatal slices in WT, IL-1αβKO and P2X7KO mice. When the slices were stimulated electrically (2 Hz) and chemically (AMPH, 30 μM), we observed a profound increase in [^3^H]DA efflux in all three genotypes (Figure 9A). Electrically induced [^3^H]DA efflux was significantly reduced in IL-1αβKO and P2X7KO mice comparison to WT mice (▪, ♦, ♦, Figure 9B). AMPH-induced [^3^H]DA efflux was attenuated by JNJ in WT and IL-1αβKO (●, ▪) but not in P2X7KO mice (Figure 9C). Furthermore, *post hoc* analyses showed that AMPH-induced [^3^H]DA release was also reduced in P2X7KO mice (♦, ♦) compared with WT and IL-1αβKO mice (Figure 9C). The absence of the P2rx7 gene significantly reduced tissue uptake compared to WT (♦) (Figure 9D).

### Chronic amphetamine treatment increased thiobarbituric acid reactive substance levels in the prefrontal cortex in wild type mice

**Figure 10:**
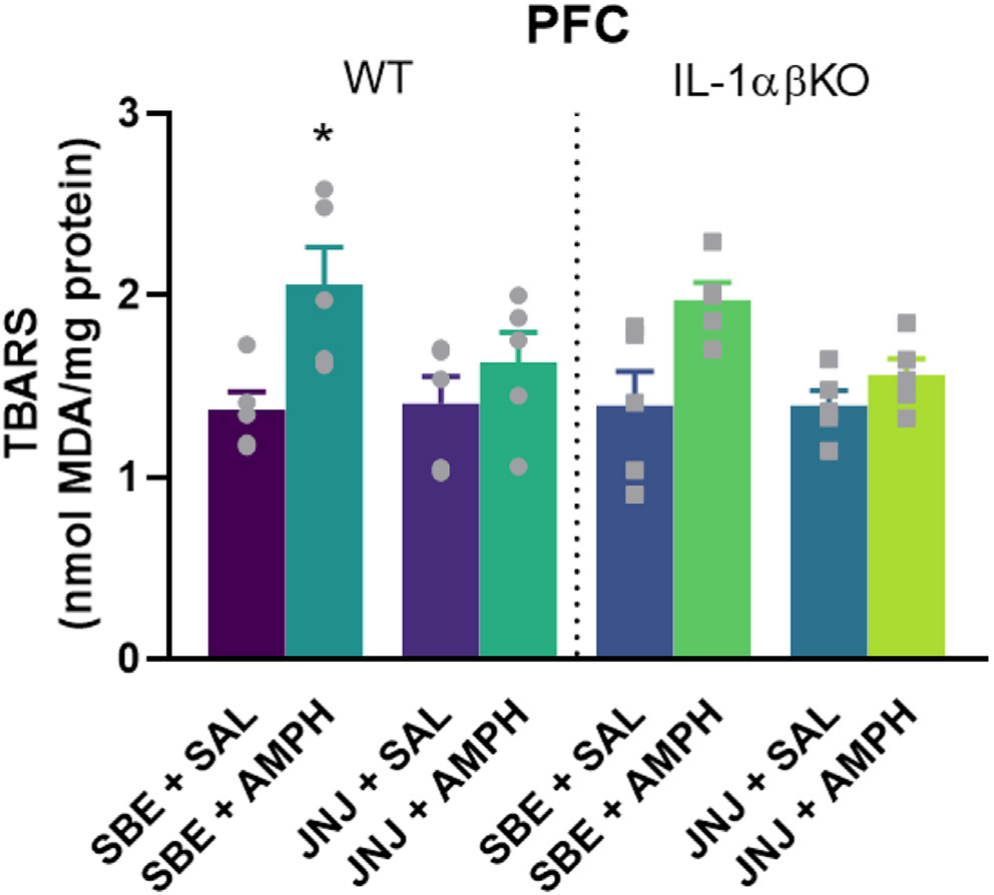
TBARS levels in the PFC 1 h after last JNJ/SBE + AMPH/SAL injection in WT and IL-1αβKO mice Values are presented as means ± SEM of n = 5 mice per group. two-way ANOVA followed by Tukey’s multiple comparison *post hoc* test. ∗p < 0.05 compared to SBE + SAL treated group in a same genotype. AMPH, d-amphetamine, IL-1αβKO, interleukin-1α/β knockout mice, JNJ, JNJ-47965567, MDA, malondialdehyde, PFC, prefrontal cortex, SAL, saline, SBE, beta cyclodextrin sulfobutyl ether, TBARS, thiobarbituric acid reactive substances, WT, wild-type mice.

TBARS levels are considered as an indicator of oxidative stress. There was a significant increase in TBARS levels in the PFC in the SBE + AMPH group in WT mice when compared to the SBE + SAL group, but not in the group treated with JNJ (Figure 10). In IL-1αβKO mice, AMPH had no effect on TBARS levels in the PFC.

### Amphetamine -induced hyperlocomotion in interleukin-1αβ knockout and wild type female mice

**Figure 11:**
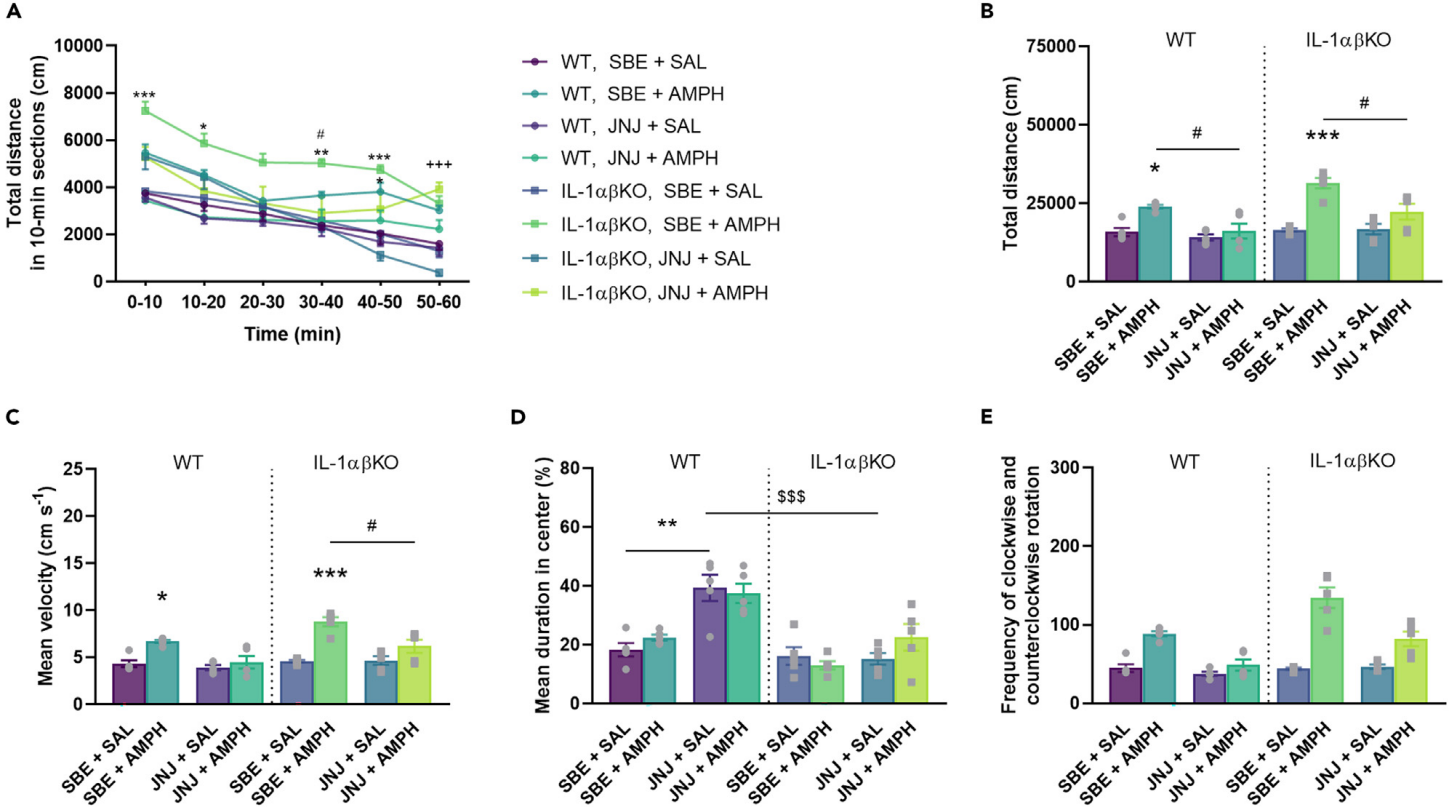
AMPH significantly induced hyperlocomotion in WT and IL-1αβKO female mice Mice were pre-treated with SBE/JNJ and 30 min later with AMPH/SAL once a day for 7 consecutive days. Immediately after the last AMPH injection, mice were subjected to the open field apparatus. (A) Distance traveled by time (time × treatment × genotype: F[15,160] = 2.619, p = 0.001487). (B) Total distance traveled. (C) Velocity. (D) Cumulative duration in central zone. (E) Circling behavior of mice. Weight % of baseline. Values are presented as means ± SEM of n = 5 mice per group. Repeated Measures ANOVA (A) followed by Tukey’s multiple comparison *post hoc* test and two-way ANOVA after square-root transformation (B, C, D) followed by Tukey’s multiple comparison *post hoc* test, and Kruskal-Wallis test with multiple comparisons (E): ∗p < 0.05, ∗∗p < 0.01, ∗∗∗p < 0.001 compared to SBE + SAL treated group in a same genotype; ^#^p < 0.05 compared to the SBE + AMPH treated group in a same genotype; ^+^p < 0.05 compared to JNJ + SAL treated group in a same genotype; ^$$$^p < 0.001 compared to the same treatment group with WT. AMPH, d-amphetamine, IL-1αβKO, interleukin-1α/β knockout mice, JNJ, JNJ-47965567, SAL, saline, SBE, beta cyclodextrin sulfobutyl ether, WT, wild-type mice.

The same treatment protocol was used as for male mice. There was no difference in total distance between WT and IL-1αβKO male and female mice, regardless of treatments (Kruskal-Wallis test: H[15,N = 120] = 66.30174, p = 0.00001; Figures 3B and 11B). Repeated SBE + AMPH (●, ▪) significantly increased total distance, mean velocity in female mice of both genotypes compared to the SBE + SAL (●, ▪) groups, which was attenuated by JNJ pre-treatment (Figures 11B and 11C). JNJ-treated IL-1αβKO female mice spend less time in the center than WT mice (Figure 11D). Circling behavior of female mice was not altered after treatments compared to the SBE + SAL groups (Figure 11E). Repeated treatments did not cause any changes in the body weight of female mice (See Figure S6).

## Discussion

It is increasingly recognized that inflammation and neuroinflammation play a key role in the development of mental disorders.^5^^,^^38^^,^^39^ The purinergic system is a major driver of inflammation. Activation of P2 receptors modulates the release of cytokines and chemokines^18^^,^^40^^,^^41^ and interacts with other neurotransmitter systems involved in mood disorders.^16^^,^^42^ We have now investigated the role of IL-1α/β and its modulation by P2X7R in an AMPH-induced mouse model of mania. To explore the endogenous P2X7R- and IL-1α/β-mediated actions, we used a P2X7R-selective antagonist, JNJ-47965567 and IL-1α/β-gene-deficient mice.

One aspect of mania is locomotor activation, which is easily measured in rodents. The usual agent for the pharmacological induction of manic hyperactivity has been the “gold-standard” AMPH.^12^^,^^43^ AMPH sensitization-induced hyperactivity has been reversed in rodents with lithium and several anticonvulsants with mood-stabilizing properties (valproate, lamotrigine, carbamazepine), indicating adequate predictive validity for this model.^44^^,^^45^ In our experiment, valproate treatment also reversed AMPH-induced hyperactivity in WT mice, without a sedative effect (See Figure S1).

We also showed that subchronic AMPH caused increased locomotion, which was alleviated by JNJ (Figure 3). This result confirms and extends previous findings showing an inhibitory effect of P2X7R blockade in acute and subchronic AMPH-induced hyperactivity models.^19^^,^^21^^,^^24^ As a primary novel finding of the study, this inhibitory effect was independent of IL-1α/β, as the effect maintained in IL-1αβKO mice.

Proinflammatory cytokines are considered to be important mediators in mental disorders.^38^^,^^46^ To investigate the effects of AMPH on the anti- and proinflammatory cytokine levels, we measured the levels of IL-1β, IL-4, IL-6, IL-10, IFN-γ, and TNF-α in brain samples after AMPH administration. LPS was used as a positive control to monitor the induction of cytokine response (See Figures 1 and S2). We found that the repeated administration of AMPH did not induce a robust systemic inflammatory response in IL-1β, IL-4, IL-6, and IFN-γ levels in the PFC, STR, and HP of wild-type and IL-1αβKO mice. However, following AMPH treatment, IL-10 levels were elevated in the PFC of WT and IL-1αβKO mice, which was reduced by JNJ treatment in WT mice. Furthermore, TNF-α levels were also increased in the PFC and STR of IL-1αβKO mice and in the STR of WT mice, which was decreased by JNJ treatment (see Figures 4 and 5). Taken together, these results suggest that repeated AMPH administration does not elicit a robust systemic inflammatory response and that the inhibitory effect of P2X7R antagonists on AMPH-induced hyperactivity involves different mechanisms. These results are in line with previous studies that have not shown AMPH-induced IL-1β induction in the brain.^47^^,^^48^ However, some studies found increases in IL-1β,^19^ IL-6 ^49^, IL-10 ^49^ and TNF-α^19^^,^^49^ or IL-17.^50^ Of note, IL-10 and TNF-α were also increased in our study. Although, like others, we did not get an increase in IL-6 levels^19^ in mice brain. Our results also support recent meta-analyses of human studies that have shown elevated levels of peripheral C-reactive protein and TNF-α, but not IL-1β, in patients with BD.^51^ Consistent with our results, a double-blind, multicentre, randomized controlled trial (NCT02902601) in patients with major depressive disorder also reported no difference in peripheral blood IL-1β levels after treatment with JNJ-57417446, another P2X7R antagonist, compared with placebo, which may reflect multiple pathways of IL-1β release that do not involve P2X7Rs,^27^ although JNJ-57417446 reduced *ex vivo* IL-1β release from LPS-stimulated peripheral leukocytes in the presence of the P2X7R agonist BzATP.^27^ Nevertheless, as AMPH-induced hyperactivity models represent only one phenotypic aspect of the manic episode within BD, our results do not exclude the involvement of other inflammatory mechanisms (e.g., TNF-α, IL-10) in mood disorders, either as a cause, consequence or a concomitant alteration.

The lack of involvement of IL-1β in the attenuating effect of JNJ on AMPH-induced hyperlocomotion was further supported by the following results: (i) the AMPH administration protocol used in our experiments did not induce significant changes in IL-1β levels in the brain regions studied (Figure 4), (ii) neither pharmacological blockade of IL-1Ra with anakinra (Figure 6), (iii) nor the downstream signaling pathway from P2X7Rs, i.e., the NLRP3 inflammasome by MCC950 (Figure 7), attenuated AMPH-induced hyperactivity.

Interestingly, examining only the behavior of naive mice, IL-1αβKO mice showed higher levels of locomotion than wild-type mice, while exhibiting similar levels of anxiety (See Figures 1, S3, and S4). This increased locomotor activity was abolished in IL-1αβKO mice when treated with SBE + SAL, and the absence of IL-1α/β did not affect hyperlocomotion following AMPH treatment. A possible explanation of this discrepancy could be that 7 days of handling and treatment abolished this difference. These results suggest that although endogenous IL-1β may have some depressant effect on locomotion, which was attenuated by gene deficiency, the behavioral stimulant effect of AMPH is largely independent of this cytokine.

While AMPH-induced hyperactivity may be a relatively valid model for one facet of mania, BD also includes several other behavioral components. One of the manic symptoms is provocative, intrusive, or aggressive behavior, which can be objectively measured and tested in mice.^43^ We therefore characterized the effect of the deletion of the IL-1α/β and P2rx7 gene on the aggressive behavior in adult mice (Figure 8). In the resident-intruder test, P2X7KO mice showed reduced interaction after 4 weeks of solitude, for both social and aggressive interactions, while the interactions of IL-1αβKO mice were not different from those of WT mice. These results are consistent with a previous study evaluating aggressive behavior using a different paradigm, which showed that P2X7KO mice exhibited less aggressive biting behavior.^52^

Monoamine transporters are critical molecular targets of medications or drugs of abuse. AMPH binds to the DA transporter and increases extracellular levels of DA. In release experiments, DA is released either in response to electric stimulation in an extracellular [Ca^2+^]-dependent manner by action potentials, or in response to AMPH due to reversal of the DA transporter in the striatum in all three genotypes. Extending previous findings,^21^ pharmacological blockade of P2X7Rs using JNJ reduced AMPH-induced DA release in WT and IL-1αβKO, but not P2X7KO mice (Figure 9). Thus, IL-1α/β is not involved in the P2X7R-mediated modulation of AMPH-induced striatal DA release.

According to the amine neurotransmitter hypotheses, BD is the result of an imbalance of neurotransmitters in the brain. In addition to its effect on DA, AMPH also affects monoaminergic pathways, increasing extracellular NA and 5-HT, and may therefore play a role in the behavioral effects of AMPH-like stimulants.^53^^,^^54^ P2X7R plays an important role in modulating the release and uptake of various neurotransmitters dysregulated in mania and depression.^22^^,^^55^ We have previously shown that neither the presence of P2X7R nor a single dose of AMPH affected 5-HT, DA and NA content in the amygdala.^21^ Others have shown that 7 days of AMPH treatment reduced 5-HT and glutamate levels in both hippocampal and striatal structures, but the use of non-selective P2X7R antagonist BBG did not alter this effect.^24^ In contrast, our current data show that although subacute AMPH administration did not significantly alter DA, NA, and 5-HT content in the PFC, STR, HP, and plasma of WT and IL-1αβKO mice, JNJ treatment did (See Figures S7 and S8). These results suggest that P2X7R blockade may regulate amine neurotransmitter levels in certain brain regions, but this depends on the application protocol.

Previous studies have identified increased oxidative stress markers in BD.^56^^,^^57^ It is well-established that P2X7R activation stimulates multiple signaling pathways such as ROS, pro-inflammatory cytokines, and nitric oxide releases in microglia and other cell types, increasing neuroinflammation and redox imbalance in the brain under chronic signals.^58^ P2X7R activation also involves the production and release of leukotriene B4 (LTB4).^59^ ROS and LTB4 release and subsequent activation of the NLRP3 complex and IL-1β release, and NLRP3 activation by LTB4 depended on ROS induction.^60^^,^^61^ Previous studies had already shown increasing lipid peroxidation levels (as an indicator of ROS) in an AMPH-induced animal model of mania^62^ and that A438079 P2X7R antagonist modulates TBARS (thiobarbituric acid reactive substances) levels.^19^ We observed that chronic AMPH treatment induced an increase in TBARS levels in the PFC of WT mice (see Figure 10), but not in IL-1αβKO mice. It has been previously observed that IL-1β can directly or indirectly induce lipid peroxidation via reactive oxygen species formation.^63^ After JNJ pretreatment AMPH did not elevate TBARS levels in WT and IL-1αβKO mice confirming the regulatory role of P2X7R in ROS production. However, whether this effect is an underlying mechanism of the inhibition of AMPH induced hyperactivity awaits further investigation.

BDNF is one of the most studied growth factor in the brain, which may have anti-inflammatory effects and mediate mood symptoms. Human studies provide conflicting data on BDNF levels in patients with BD.^64^^,^^65^ In rodents, AMPH treatment may induce changes in BDNF levels.^19^^,^^66^ Although we were unable to assess these changes in our study (See Figure S9), our results are in agreement with another studies showing that repeated low-dose AMPH administration had no effect on BDNF levels in the HP and STR.^67^^,^^68^ Consistent with the literature, the blockade of P2X7R did not result in any action on BDNF levels, suggesting other microglial P2 receptors are involved in this process.^19^

BD affects both women and men. The prevalence of BD in the general population has increased over the past decade, but few epidemiological studies are available on the gender distribution of BD, so the question remains unanswered: could the frequently reported increase in BD diagnosis be sex-specific^69^? We already know that the clinical presentation of patients with BD shows sex differences, especially in sub-phenotypes related to the frequency of mood episodes, such as rapid cycling. In a small number of human studies, they concluded that the effect of P2RX7 variants on BD may be sex-specific, and that increased P2X7 activity can potentially increase the risk of BD in women.^70^ In the present study, we used mainly male mice, but we examined the effect of AMPH treatment in WT and IL-1αβKO female mice and the effect of P2X7 antagonist on hyperlocomotion in females in the open field test (see Figure 11). Previous study showed that AMPH administration induced higher locomotor activity in female than in male mice.^71^^,^^72^ In contrast, our results show that AMPH-induced hyperlocomotion was not different in WT and IL-1αβKO male and female mice (Figures 3 and 11). However, as in male mice, pretreatment with JNJ in WT and IL-1αβKO females reduced the locomotion enhancing effect of AMPH independently of IL-1α/β. Although we obtained this result, it is worth considering the sex-specific effects of future research investigating the effect of P2RX7 on BD.

### Conclusions

The main novelty of our study is that the effect of P2X7R inhibition to alleviate AMPH-induced hyperlocomotion is independent of the canonic IL-1β-NLRP3 signaling pathway; and IL-1β plays a limited role in mediating AMPH-induced hyperactivity in rodents. Rather, the receptor may be involved in the regulation of uptake and release of monoamines and neuroinflammation directly or through the production of TNF-α, IL-10 and ROS, especially in brain regions activated during hyperlocomotion. Furthermore, we show the effects of AMPH-induced hyperactivity in IL-1β-deficient mice and NLRP3 inhibition *in vivo*, and the effects of an identical amphetamine treatment on the inflammatory response of the brain. Therefore, our results provide a better understanding of the mechanism of action of P2X7R inhibition and AMPH in animal models of mania and suggest some relevant pathways.

### Limitation of the study

Some limitations of the present study should be noted. First, mania is more than hyperactivity, and locomotor activity measurement should not be the only preclinical outcome measure. In addition, hyperactivity is seen in other neuropsychiatric disorders. Furthermore, the specific mechanisms that mediate the involvement of IL-1β in AMPH-induced behavior needs further investigations.

Second, IL-1β levels were also measured in brain areas, not only in plasma. Before collection, the brain was not perfused, so tissue IL-1β levels may be partly derived from the blood rather than from endogenous production by the tissue itself. Presumably, this does not significantly affect IL-1β levels in the brain, as the same methodology was used for brain sample collection for all treatments/experiments without exception.

Third, IL-1β levels in the PFC of wild-type mice tended to double after AMPH treatment compared to controls (Figure 4A), although this difference was not statistically significant. Additionally, an increase in TBARS levels was also observed in the PFC of WT and IL-1αβKO mice after AMPH treatments, but was only significant in WT mice (Figure 10).

Fourth, anakinra, even if only slightly, enhanced the effect of AMPH treatment compared to the SAL+AMPH group (Figure 6A).

In light of these results, we cannot completely rule out a P2X7R independent role for IL-1β in mania, which awaits further investigations.

## STAR★Methods

### Key resources table

**Table:** 

REAGENT or RESOURCE	SOURCE	IDENTIFIER
**Antibodies**		

IFN-γ	BD Biosciences	RRID:AB_2869141
IL-1β	BD Biosciences	RRID:AB_2869322
IL-4	BD Biosciences	RRID:AB_2869143
IL-6	BD Biosciences	RRID:AB_2869146
IL-10	BD Biosciences	RRID:AB_2869145
TNF-α	BD Biosciences	RRID:AB_2869144

**Chemicals, peptides, and recombinant proteins**		

D-amphetamine hemisulfate salt	Sigma-Aldrich	Cat# A5880
Valproic acid sodium salt	Sigma-Aldrich	Cat# P4543
JNJ-47965567	Tocris	Cat# 5299
Beta cyclodextrin sulfobutyl ether	Captisol	Cat# RC-0C7-100
MCC950	Tocris	Cat# 5479
Anakinra/Kineret 100 mg/0.67 mL, SOBI	local pharmacy	N/A
LPS	Sigma-Aldrich	Cat# L2880
[3H]DA	ARC Inc	N/A
Isofluran CP	Medicus partner Inc	N/A

**Critical commercial assays**		

Mouse IL-1β/IL-1F2 Quantikine ELISA Kit	R&D Systems	Cat# MLB00C
BCA Protein Assay Kit	Thermo Scientific	Cat# 23227
Cytometric Bead Array Kit	BD Biosciences	Cat# 558267; RRID:AB_2869126
Human/Mouse BDNF DuoSet ELISA Kit	R&D Systems	Cat# DY248
Lipid Peroxidation (MDA) Assay Kit	Sigma-Aldrich	Cat# MAK085

**Deposited data**		

Raw data	Mendeley	https://doi.org/10.17632/dfk676gdzt.2

**Experimental models: Organisms/strains**		

Mouse: C57BL/6J	local animal house	JAX: 006494
Mouse: IL-1αβ double-knockout	local animal house, Original owners of line: Stuart M. Allan, Emmanuel Pinteaux	N/A Horai et al.^73^
Mouse: P2X(7)R knockout, B6.129P2-P2rx7tm1Gab/J	local animal house, Original owner of line: Christopher Gabel, Pfizer	MGI:J:66835 RRID:IMSR_JAX:005576

**Software and algorithms**		

EthoVision XT 13.0	Noldus	RRID:SCR_000441
FCAP ARRAY V3	BD Biosciences	Cat# 652099
STATISTICA V 14.0.1	TIBCO Software Inc	https://docs.tibco.com/products/tibco-statistica-14-0-1
GraphPad Prism 8.0	GraphPad Software	RRID:SCR_002798
G∗Power 3.1	Heinrich Heine Universität Düsseldorf	RRID:SCR_013726

### Resource availability

#### Lead Contact

Further information and requests for resources should be directed to and will be fulfilled by the Lead Contact, Beáta Sperlágh (sperlagh@koki.hu).

#### Materials availability

This research project did not generate new unique reagents and techniques.

#### Data and code availability

• Raw data have been deposited at Mendeley and are publicly available as of the date of publication. The DOI is listed in the key resources table.
• This paper did not generate new codes.
• Any additional information required to reanalyse the data reported in this paper is available from the lead contact upon request.

### Experimental model and study participant details

#### Animals

In the experiments, male and female WT (C57BL/6J) and IL-1α/β knockout mice (weight 20–27 g) were used. WT, IL-1α/β (original breeding pairs from Yoichiro Iwakura, Tokyo University of Science, Japan) and P2X7R knockout mice (from Christopher Gabel, Pfizer) were bred and genotyped in the MGTU of IEM (Medical Gene Technology Unit of Institute of Experimental Medicine) as described in previous studies.^73^^,^^74^ Animals were maintained on a 12:12 light-dark cycle in a temperature- (23 ± 2°C) and humidity-controlled room (60 ± 10%), with food and water *ad libitum*. Before and under the behavioral experiments, up to 5 adult littermates mice per cage were housed in standard mouse cages with corncob bedding (expect for residents in the resident intruder test). To enrich the environment, cardboard bedding material and tubes were placed in each cages. All behavioral experiments were conducted between 8:00 a.m. and 2:00 p.m. Behavioral tests were performed in an experimental room after 30 min of habituation. To confirm the inactivity of IL-1β in the adult brain, we characterized the phenotype of IL-1αβKO mice in response to behavioral and LPS (1 or 20 mg kg^−1^) challenge by measuring IL-1β using multiplex bead array assay or ELISA assay (See Figure 1; Figure S2).

The experimental procedures described in this manuscript have been approved by the local Animal Care Committee of the IEM HAS (PE/EA/297-1/2021) and followed the guidelines of Hungarian Act of Animal Care and Experimentation guidelines (40/2013, II.14), which in line with the Directive 2010/63/EU. Animals were treated humanely, and every effort was made to minimise animal suffering and reduce the number of animals used in experiments. Animal experiments were reported in accordance with ARRIVE 2.0 guidelines.^75^ The exact number of mice in each experimental group is given in the legend of corresponding figures.

#### Chemical compounds

The following chemicals were used: the CNS stimulant d-amphetamine hemisulfate salt (AMPH, PubChem CID: 24891028, Cat# A5880, Sigma-Aldrich, St. Louis, MO, US), the histone deacetylase inhibitor valproic acid sodium salt (VAL, PubChem CID: 16760703, Cat# P4543, Sigma-Aldrich, St. Louis, MO, US), the brain-penetrant potent and selective P2X7R antagonist JNJ-47965567 (JNJ, PubChem CID: 66553218, Cat# 5299, Tocris, Minneapolis, MN, US) and its vehicle beta cyclodextrin sulfobutyl ether (SBE, PubChem CID: 131634907, flat, Cat# RC-0C7-100, Captisol, San Diego, CA, US), the potent NLRP3 inflammasome inhibitor, MCC950 (MCC, PubChem CID: 91826093, CRID3 sodium salt, Cat# 5479, Tocris, Minneapolis, MN, US), the IL-1 receptor antagonist (IL-1Ra) anakinra (ANA, Kineret, Swedish Orphan Biovitrum AB, SOBI, Stockholm, Sweden). LPS was purchased from Sigma-Aldrich (O55:B5, Cat# L2880, St. Louis, MO, US). Other materials used for experiments were obtained from general commercial resources and were of the highest grade.

### Method details

#### Drug administration and experimental setup

The pharmacological animal model of AMPH-induced mania was adopted from a previous study^24^ and performed as follows: mice received intraperitoneal (i.p.) injections of AMPH (2 mg kg^−1^) or saline (0.9% NaCl) once daily for eight consecutive days. The low dose of AMPH was chosen to affect primarily the locomotor activity of the mice.^76^ To validate the model, VAL (200 mg kg^−1^) was administered 30 min before each AMPH or saline injection for eight consecutive days. The dose of VAL was chosen based on our previous experience and the literature.^77^^,^^78^ Validation of the model is shown in Figure S1.

Mice were also given i.p. injections of JNJ (30 mg kg^−1^), MCC (25 mg kg^−1^) and ANA (100 mg kg^−1^) or vehicle (SBE) or saline for eight consecutive days, 30 min before the respective AMPH or saline injection. The dose of JNJ and MCC were chosen based on our previous studies and the literature.^20^^,^^79^ High dose i.p. injections of ANA were used because it has been shown that therapeutic concentrations in the brain are low and can only be achieved by peripheral injection of a high dose.^80^^,^^81^ No behavioral tests were performed between days 1 and 6. On the seventh day, immediately after the last injection, the mice were subjected to an open field test. After the behavioral tests, on the eighth day, 1 h after AMPH treatment, the animals were euthanized by an anesthetic overdose of the isoflurane inhalation in accordance with the guidelines of Directive 2010/63/EU.

For LPS challenge, a single i.p. injection of 1 or 20 mg kg^−1^ LPS was administered and IL-1β concentrations were measured in plasma and brain samples of mice (See Figures 1 and S2). Since the 1 mg kg^−1^ dose induced only modest IL-1β elevations (See Figure S2), a high dose (20 mg kg^−1^) was also chosen to validate the genetic model, which can induce more severe endotoxic shock and systemic inflammation. Plasma samples were taken 6 h after treatment, as IL-1β levels in mouse serum is already significantly elevated at this time.^82^ The LPS-injected mice were clearly sick at this time-point, although they all survived. After termination, blood, brain structures were collected simultaneously for further analysis. In the [^3^H]DA release experiments naive mice were used.

#### Open field test

In order to investigate the effect of AMPH on mania-like symptoms in mice, such as exploratory behavior and activity level, we conducted an open field test as previously described.^19^ All 6–11 week old animals were placed individually in the center of the arena (40 × 40 × 20 cm, covered by acrylic sheet) and left free for 60 min. The animal’s behavior was recorded and analyzed using the EthoVision XT 13.0 video tracking system (RRID:SCR_000441, Noldus Information Technology, Wageningen, Netherlands). The apparatus was cleaned after each trial with 20% ethanol and water.

#### Elevated plus maze test

The elevated plus maze combines a natural preference for dark spaces with an aversion to lighted, open and/or elevated areas. The delay of entry into the arms, the time spent inside and the number of entries into each type of arm are used to determine the level of anxiety or risk-taking behavior.^83^^,^^84^^,^^85^ A plexiglas plus-shaped maze consisting of two dark and enclosed arms and two open and lit arms, raised 50 cm above the ground, was used to study anxiety-related behavior. The arms were 30 × 7 cm with a center area of 7 × 7 cm and the walls of the closed arms were 30 cm high. Individual mice were placed in the center of the maze, facing to the closed arm, tracked with a video camera for 5 min, and then returned to their home cage. The plus maze was cleaned with a 20% alcohol solution between trials. Time spent in the maze and frequency of visits to different zones of the maze were scored using EthoVision XT 13.0 (Noldus).

#### Resident-intruder test

In order to investigate the mania-like symptoms in mice, such as aggressive behavior, a resident-intruder test was performed as described previously with slight modifications.^43^ Resident male mice (10-week-old at the test day) were housed individually for 4 weeks in standard home cages in a room with a reversed light-dark cycle. The intruder mice (8-week-old at the test day) were grouped in the same colony room 2 weeks before the test. The intruders were adults but younger than the residents. All tests were conducted in the dark hours. For the test, the resident and intruder mice were transferred in their home cages to an experimental room the day before the test. All items were removed from the resident cage 10 min before the test. A male intruder mouse was introduced into the home cage of a resident male and their behavior was digitally recorded from the side of the cage for a 10 min.

Recordings were analysed manually to score aggressive interactions. The frequency and duration of behaviors were analyzed during the test from the video. The observer was blinded to the genotype of the mice. Behaviors analyzed included aggressive (aggressive grooming, upright posture, boxing, chasing, tail rattling, clinch, wrestling, dominant posture, keeping down) and social contacts (sniffing, allogrooming, following). The resident’s aggression scores were calculated as the ratio of aggressive interactions out of total (aggressive + non-aggressive) interactions. At the end of the test, the intruder mice were removed and placed back in their home cages. Each resident mouse was tested only once. Intruders were used twice with an hour break.

To minimize harm to the animals, the mice were briefly separated when the attacks became vicious and included significant biting. Therefore, the total duration of the attacks could not be scored and only the number of aggressive and non-aggressive interactions were scored from the recordings.

#### IL-1β assay

In order to validate the IL-1αβ gene deficiency in mice, plasma and brain IL-1β levels were examined under basal conditions and after endotoxin challenge. WT and IL-1αβKO mice were treated i.p. with 20 mg kg^−1^ LPS. Six hours after LPS injection, mice were euthanized by an anesthetic overdose of the isoflurane inhalation and approx. 800 μL blood and brain structures were collected. The brain was not perfused before tissue harvesting. Blood was taken from the vena cava with heparinized syringes inserting a 25 G × 5/8″ needle. PFC, HP and STR were homogenized in phosphate buffered saline. The concentration of IL-1β in the brain structures was determined using a specific commercially available Mouse IL-1β/IL-1F2 Quantikine ELISA Kit (R&D Systems, Minneapolis, MN, US, Cat# MLB00C) according to the manufacturer’s instructions. Samples were not diluted. Absorbance was measured at 450 nm. IL-1β concentration was expressed as pg mg^1^ protein. IL-1β levels were normalized to total protein levels measured photometrically using the BCA Protein Assay Kit (Thermo Scientific Pierce, Rockford, IL, US, Cat# 23227). Absorbance was measured at 560 nm using a Cytation 5 Cell Imaging Multi-Mode Reader (BioTek, Winooski, VT, US).

#### Multiplex bead array analyses of cytokines

Brain and approx. 800 μL blood was taken from the vena cava with heparinized syringes inserting a 25 G × 5/8″ needle from mice deeply anesthetized with isoflurane until loss of paw withdrawal reflex. The brain was not perfused before tissue harvesting. PFC, HP and STR were homogenized in phosphate buffered saline. Subsequently, blood samples were centrifuged at 12000 rpm for 10 min at 4°C and supernatant plasma samples were collected. Concentration of anti- and proinflammatory mediators IFN-γ (RRID:AB_2869141), IL-1β (RRID:AB_2869322), IL-4 (RRID:AB_2869143), IL-6 (RRID:AB_2869146), IL-10 (RRID:AB_2869145), TNF-α (RRID:AB_2869144) were measured from plasma and brain samples using a BD FACSVerse flow cytometer, Cytometric Bead Array Kit (BD Biosciences, San Jose, CA, US, Cat# 558267). Data were analyzed using FCAP ARRAY V3 Software (BD Biosciences, San Jose, CA, US, Cat# 652099) as described previously.^86^ Cytokine levels were normalized to total protein levels measured by photometry using the BCA Protein Assay Kit (Thermo Scientific Pierce, Rockford, IL, US, Cat# 23227). Absorbance was measured at 560 nm using a Cytation 5 Cell Imaging Multi-Mode Reader (BioTek, Winooski, VT, US).

#### [^3^H]DA release

Male mice (14–18 weeks old) were anesthetized with isoflurane inhalation and subsequently decapitated. The brain was quickly removed and placed in ice-cold Krebs solution. Unless otherwise indicated, all the experiments were performed at 37°C in Krebs solution (pH 7.4) containing 113 mM NaCl, 4.7 mM KCl, 2.5mM CaCl_2_, 1.2 mM KH_2_PO_4_, 1.2 mM MgSO_4_, 25 mM NaHCO_3_, 11.5 mM glucose, 0.3 mM ascorbic acid and 0.03 mM Na_2_EDTA, continuously saturated with a 95% O_2_ + 5% CO_2_. The striatum was then harvested and cut into 400-μm-thick slices. [^3^H]DA release experiments were performed as previously described by our laboratory.^87^ Briefly, slices were incubated in 1 mL Krebs solution containing 5 μL of [^3^H]DA (1 μCi/mL, specific activity 60 Ci/mmol, ARC Inc., St. Louis, MO, US) for 45 min. Following incubation, slices were rapidly transferred to tissue chambers and perfused continuously with Krebs solution at a rate of 0.5 mL min^−1^. After 60 min of preperfusion, samples were collected every 3 min and [^3^H]DA was assayed. The release of radioactivity was stimulated over two periods (ES and AMPH) by electric field stimulation (Grass S88 stimulator with the following parameters: 40 V, 2 Hz, 1 ms, 2 min) and chemical stimulation with perfusion of 30 μM AMPH for 3 min during the 3^rd^ and 10^th^ collection. In some experiments, 100 nM JNJ was administered 16 min before the electrical field stimulation and onwards. At the end of the radioactivity collection, the residual radioactivity in the preparations was extracted with 10% trichloroacetic acid and measured using an aliquot of the supernatant.

Radioactivity in tissues and collection samples was measured using a Packard 1900 Tricarb liquid scintillation spectrometer (Packard, Canberra, Australia) and the [^3^H]DA content was normalized to the Bq g^−1^ tissue. Tissue tritium uptake was determined as the sum of release plus tissue content after the experiment and expressed in Bq g^−1^. The tritium content in the perfusate samples was expressed as a percentage of the basal level and plotted as a function of time. The effect of electric field stimulation or AMPH on the release was evaluated as the ratio of the area under the curve of the total radioactivity release to the resting release in response to the first and second stimulation, respectively (FRS).

The [^3^H]DA was purchased from Izotóp Intézet Ltd. (Budapest, Hungary) and the isoflurane for anesthesia was from Medicus Partner Ltd. (Biatorbágy, Hungary).

#### HPLC determination of biogenic amines

The PFC, HP and STR were dissected on ice and homogenized with 0.1 M PCA solution containing 10 μM theophylline (as an internal standard) and 0.5 mM sodium metabisulphite (antioxidant for biogenic amines). The concentration of brain tissue homogenate from WT and IL-1αβKO mice was 100 mg mL^−1^. Tissue extracts were centrifuged at 3510 g for 10 min at 4°C and the pellet was saved for protein measurement. Perchloric anion was precipitated from the supernatant with 4 M dipotassium phosphate and removed by centrifugation. Samples were stored at −20°C until analysis and 10 μL were used for separation. NA, DA and 5-HT were quantified using an online column switching liquid chromatographic technique. Solid phase extraction (SPE) was performed on a HALO Phenyl-Hexyl (75 × 2.1 mm I.D., 5 μm) column and coupled to an ACE Ultra Core Super C-18 (150 × 2.1 mm I.D., 5 μm) analytical column for separation. The mobile phase flow rate [“A” 10 mM potassium phosphate, 0.25 mM EDTA “B” with 0.45 mM octane sulphonyl acid sodium salt, 8% acetonitrile (v/v), 2% methanol (v/v), pH 5.2] was 250 μL min^−1^ in a step gradient application.^88^ A Shimadzu LC-2AD 0 HPLC system was used for analysis. Analytes were signaled with Agilent UV (1100 series variable wavelength) and amperometric detectors (BAS CC-4) in cascade mode. Catechol and indole amines were detected electrochemically at an oxidation potential of 0.73 V, while the internal standard was signaled by UV at 253 nm. The concentrations were calculated using the formula (Ai ∗f ∗B)/(C ∗ Di ∗E) (Ai: Area of biogenic amine component; B: Sample volume; C: Injection volume; Di: Response factor of 1 pmol biogenic amine standard; E: Protein content of sample; f: recovery factor of Internal Standard (IS area in calibration/IS area in actual)). The results were expressed as pmol mg^−1^ protein.

#### BDNF measurement

BDNF was measured using the Human/Mouse BDNF DuoSet ELISA Kit (R&D Systems, Minneapolis, MN, US, Cat# DY248) according to manufacturer’s instructions. Tissue samples were diluted 4× for BDNF measurements; results are presented as the mean of two parallels for each sample. The absorbance of BDNF was read at 450 nm. Tissue levels of BDNF were expressed as pg mg^−1^.

#### TBARS measurement

Lipid peroxidation levels were measured using the commercial thiobarbituric acid reactive substances (TBARS) assay kit. The protocol was adapted according to manufacturer’s instructions (Sigma-Aldrich, St. Louis, MO, US, Cat# MAK085). Results were expressed as nmole of malondialdehyde (MDA) mg^−1^.

### Quantification and statistical analysis

Sample size was calculated using the freely downloadable G∗Power software as described previously^89^ and estimated based on a pilot study of AMPH- and vehicle-treated mice. Mice were randomly assigned to experimental groups using an Excel protocol before the start of the experiment. After the behavioral experiment, these mice were randomly selected and used for BDNF ELISA, multiplex bead array assay (FACS) and high-pressure liquid chromatography (HPLC) measurements. Investigators blind to the experimental status of the subject performed the data acquisition and quantifications. For *in vitro* experiments, data points were derived from two parallel measurements. Statistical analysis was performed only for studies where the size of each group was at least n = 5. Note that group size is the number of independent values. Outliers were included in the data analysis and presentation. All values are presented as mean ± SEM (error bars). The normality of the distribution of the experimental data was tested using the Shapiro-Wilk normality test. Depending on the datasets, statistical analyses were performed with unpaired Student’s *t* test, two-way ANOVAs with Tukey’s *post hoc* test with or without repeated measures if the data were normally distributed, if not, Kruskal-Wallis test with multiple comparisons or Friedman test using STATISTICA version 14.0.1 software (TIBCO Software Inc., Palo Alto, CA, US). *Post hoc* tests were only performed when F in ANOVA achieved p < 0.05. p-values of less than 0.05 were considered statistically significant throughout the study.

#### Additional resources

This article contains supporting information that includes supplementary file.
