# Supplementary material for: P2X7 receptor inhibition alleviates mania-like behavior independently of interleukin-1β
Source: iScience. 2024 Feb 20;27(3):109284. doi: 10.1016/j.isci.2024.109284 (PMC10914489; doi:10.1016/j.isci.2024.109284)
Supplement: Document S1. Figures S1–S9 [file mmc1.pdf]

iScience, Volume *27*

Supplemental information

P2X7 receptor inhibition alleviates mania-like behavior independently of interleukin-1β

Flóra Gölöncsér, Mária Baranyi, Pál Tod, Fruzsina Maácz, and Beáta Sperlágh

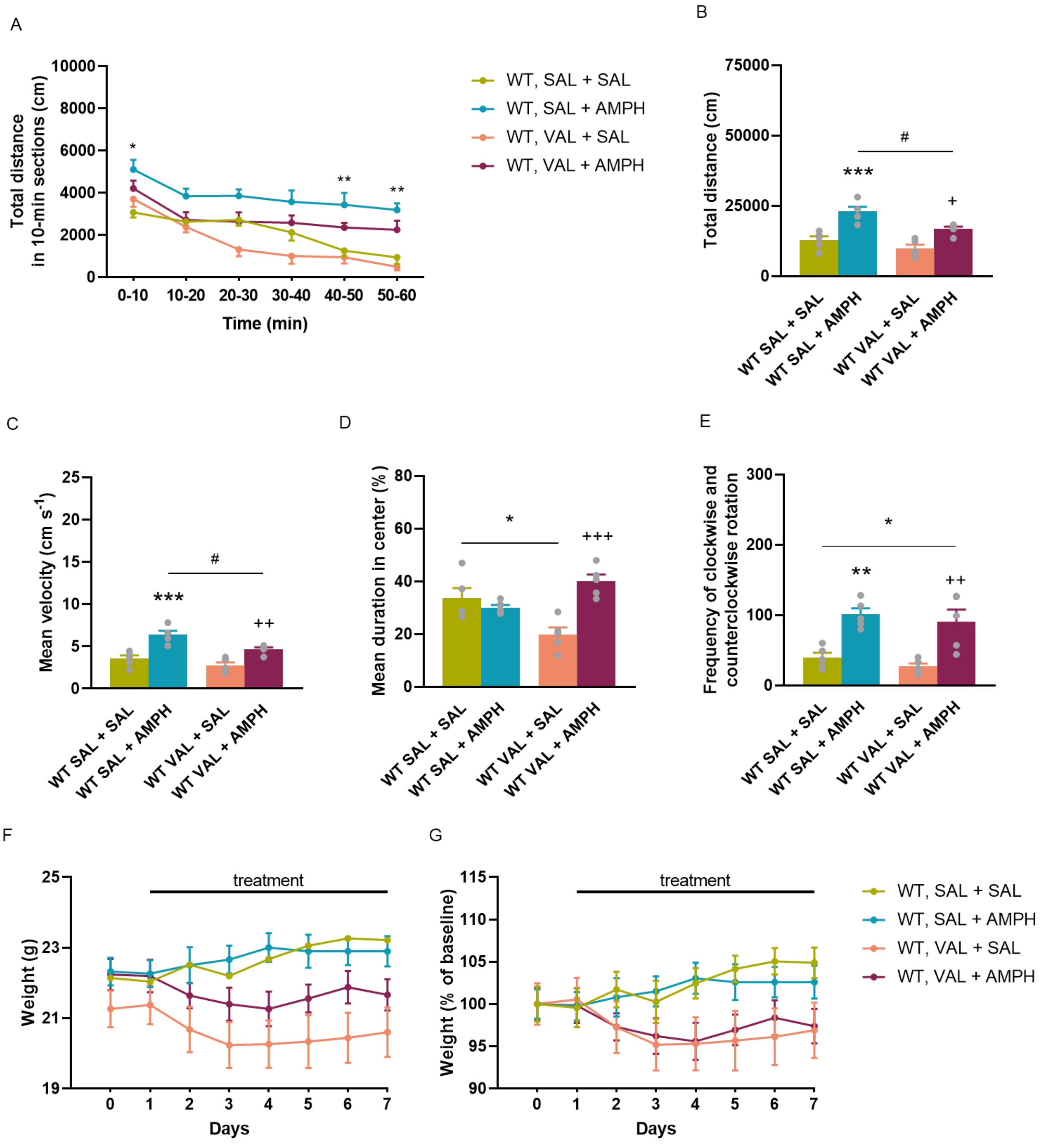

Figure S1. VAL significantly decreased AMPH-induced locomotion in WT mice, related to Figure 3. To validate our model, mice were treated with 200 $mg\ kg^{-1}$ valproic acid (VAL) 30 min before AMPH or saline treatment for 7 days, and then the locomotor activity of the mice was analysed in an open field test. The treatment and the test protocol were selected on the basis of what was previously described, with minor modifications [1-3]. Immediately after the last AMPH injection, mice were subjected to the open field apparatus. (A) Distance travelled by time (time × treatment: $F[15,80] = 1.6788$, $p = 0.0721$). (B) Total distance travelled. AMPH increased locomotion significantly in WT mice (●) which was decreased by VAL (●).VAL (●) did not affect the activity of mice compared to SAL + SAL (●). (C) Velocity. AMPH increased velocity significantly in WT mice (●) which was decreased by VAL (●). (D) Cumulative duration in central zone. Mice treated with VAL + AMPH (●) spent more time in the centrum than the VAL + SAL treated group (●). (E) Circling behaviour of mice. AMPH-treated mice (●, ●) circled the arena more than SAL-treated mice (●), and this

movement was not affected by VAL treatment (●). (F) Mice were weighed daily for 8 days. VAL induces weight loss in mice (time × treatment: $F[21,112] = 12.145$, $p = 0.0000001$). (G) Weight % of baseline. Values are presented as means ± SEM of n=5 mice per group. Repeated Measures ANOVA (A, F, G) and One-way ANOVA (B, C, D, E (after square root transformation)) followed by Tukey's multiple comparison *post hoc* test: $^{*}p < 0.05$, $^{**}p < 0.01$, $^{***}p < 0.001$ compared to SAL + SAL treated group in a same genotype; $^{\#}p < 0.05$ compared to SAL + AMPH treated group in a same genotype; $^{+}p < 0.05$, $^{++}p < 0.01$, $^{+++}p < 0.001$ compared to VAL + SAL treated group in a same genotype. AMPH, d-amphetamine, SAL, saline, VAL, valproic acid sodium salt, WT, wild-type mice.

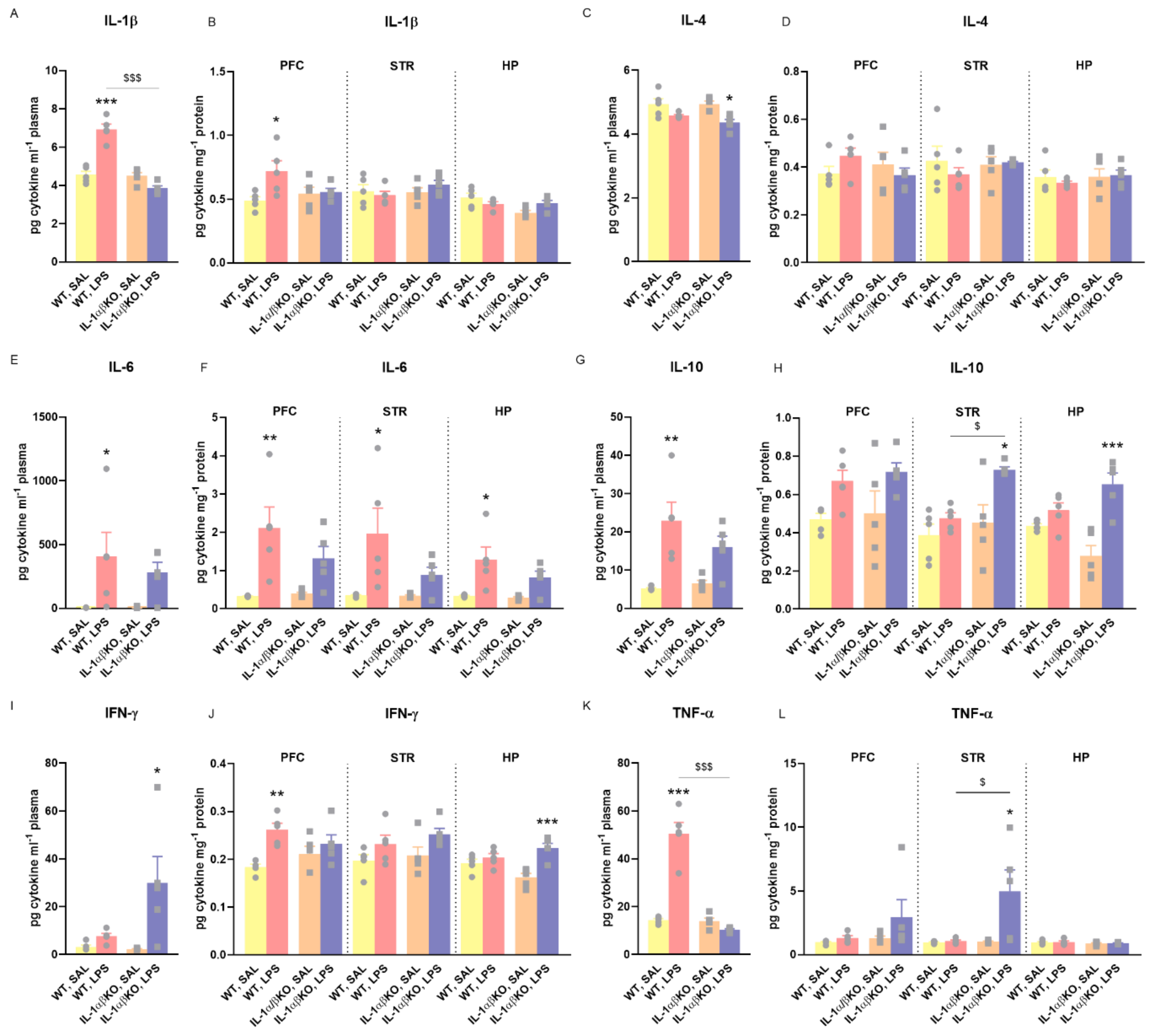

Figure S2. Inflammatory cytokine levels in the PFC, STR and HP 6 hours after saline or 1 mg kg^{-1} LPS treatment, related to Figure 1. After LPS treatment, a significant increase was observed only in plasma IL-1β levels in WT mice. Expression of pro- and anti-inflammatory cytokine levels were measured by CBA analysis in WT and IL-1αβKO mice. Values are presented as means ± SEM of n=5 mice per group. Two-way ANOVA followed by Tukey's multiple comparison *post hoc* test: *p < 0.05, **p < 0.01, ***p < 0.001 compared saline treated group in a same genotype; $^{\$}p < 0.05$, $^{\$\$\$}p < 0.001$ compared to the same treatment group with WT. IFN, interferon; IL, interleukin; IL-1αβKO, interleukin-1α/β knockout mice, LPS, lipopolysaccharides, TNF, tumor necrosis factor, WT, wild-type mice.

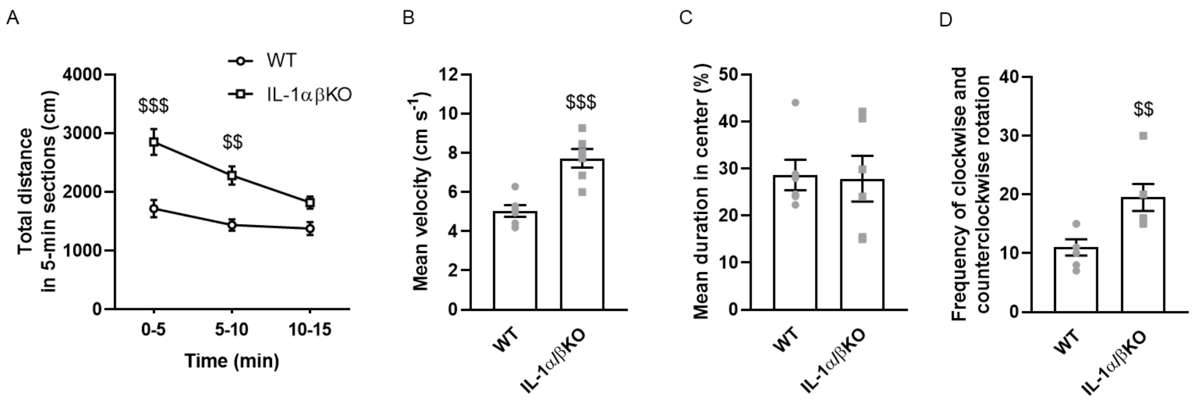

Figure S3. IL-1αβKO were more active in the open field test than WT mice, related to Figure 1. Mice were placed in the centre of the arena and left free for 15 min. (A) Distance travelled by time. Mice were placed in the centre of the arena and left free for 15 min. (B) Velocity. (C) Cumulative duration in central zone. (D) Circling behaviour of mice. Values are presented as means ± SEM of n=6 mice per group. Repeated Measures ANOVA followed by Tukey's multiple comparison *post hoc* test (A), Student's *t* test (B, C, D): $^{\$\$}p < 0.01$, $^{\$\$\$}p < 0.001$ compared to WT mice. IL-1αβKO, interleukin-1α/β knockout mice, WT, wild-type mice.

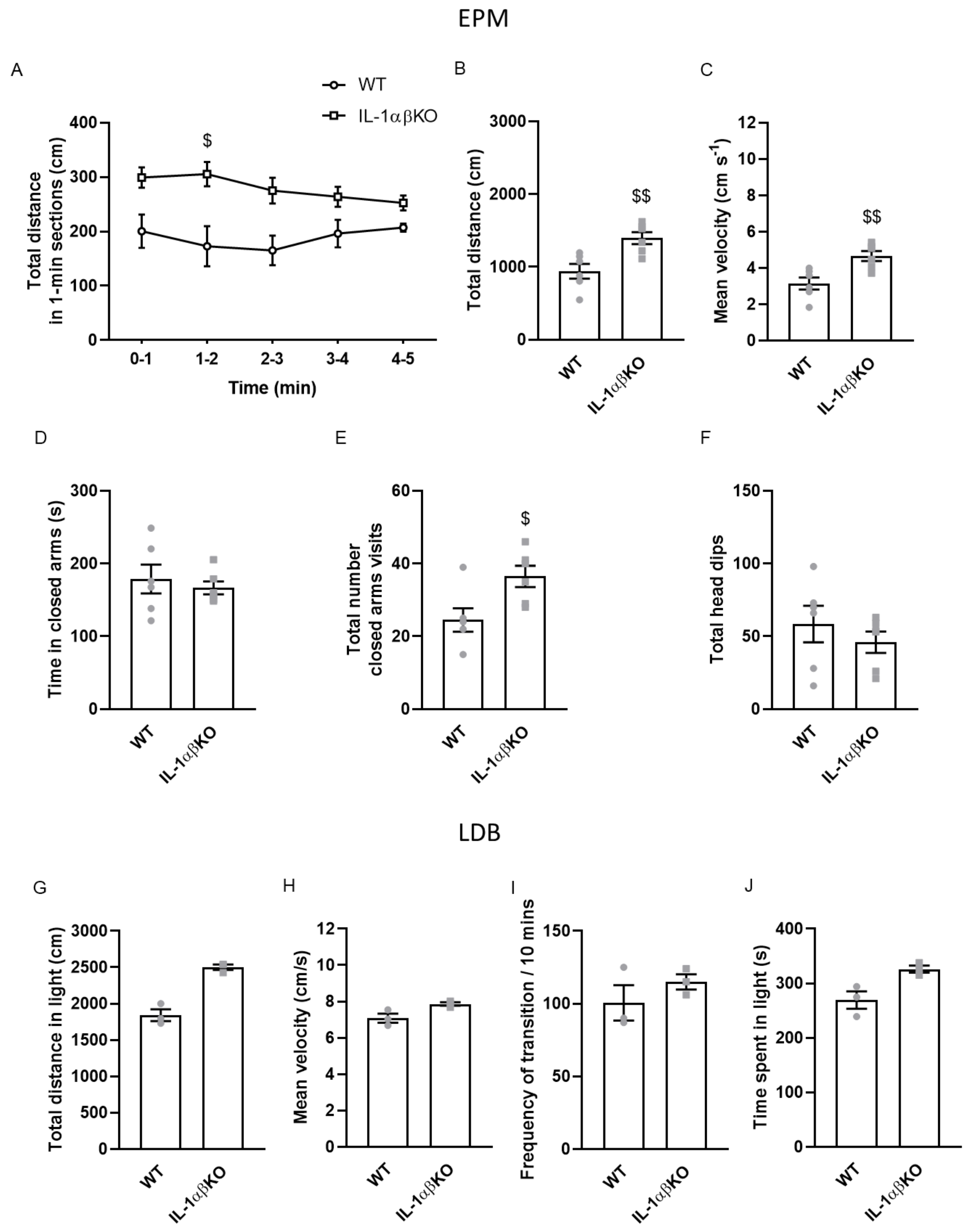

Figure S4. IL-1αβKO were not more anxious in the elevated plus maze (EPM) and light-dark box (LDB) tests than WT mice, related to Figure 1. IL-1αβKO were more active in the EPM test than WT mice. (A) In the EPM, mice were placed in the centre of the arena and left free for 5 min. Distance travelled by time. (B) Total distance travelled. (C) Velocity. (D, E) Cumulative duration in closed arms and total number of closed arms visits. (F) Total head dips during the test in naïve males. (G) In the LDB, mice were placed in the light box of the arena and left free for 10 min. Distance travelled by time in light. (H) Velocity. (I) Frequency of light-dark transitions. (J) Cumulative duration in light. Values are presented as means ± SEM of n=6 mice per group. Repeated Measures ANOVA (A) followed by Tukey's multiple comparison *post hoc*

test and Student's *t* test (B, C, D, E, F): $^{\$}p < 0.05$, $^{\$\$}p < 0.01$ compared to WT mice. EPM, elevated plus maze, IL-1αβKO, interleukin-1α/β knockout mice, LDB, light-dark box, WT, wild-type mice.

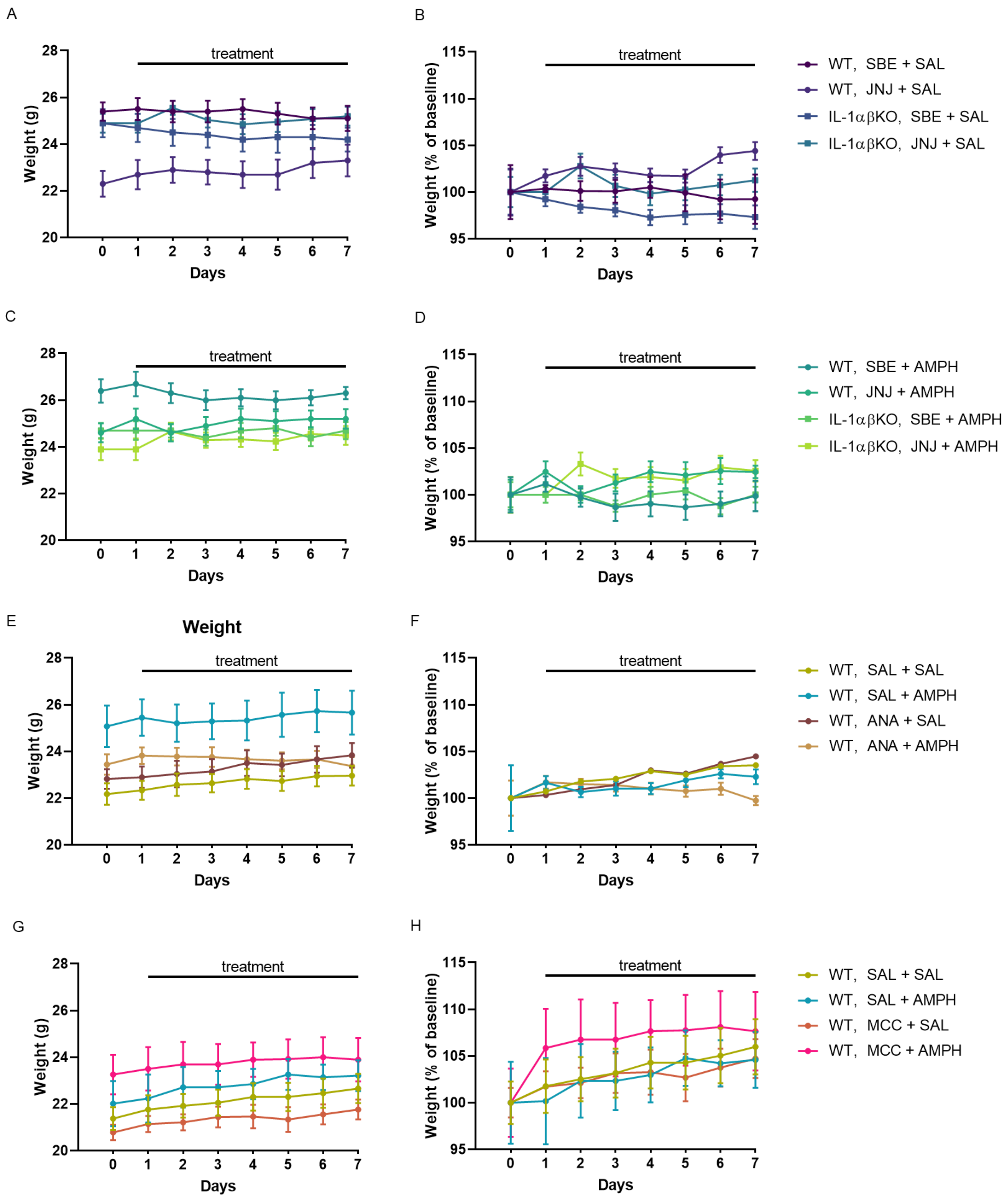

Figure S5. Treatments did not cause any changes in the body weight of mice, related to Figure 3, Figure 6 and Figure 7. Pretreatments and AMPH treatment did not affect the weight of the mice. (A, C, E, G) Mice were weighed daily for 8 days. (B, D, F, H) Weight % of baseline. Values are presented as means ± SEM of n=5-10 mice per group. Repeated Measures ANOVA (A, E, F, G, H) followed by Tukey's multiple comparison *post hoc* test and Friedman's test (B, C, D). AMPH, d-amphetamine, ANA, anakinra, IL-1αβKO, interleukin-1α/β knockout mice, JNJ, JNJ-47965567, MCC, MCC 950, SAL, saline, SBE, beta cyclodextrin sulfobutyl ethers, WT, wild-type mice.

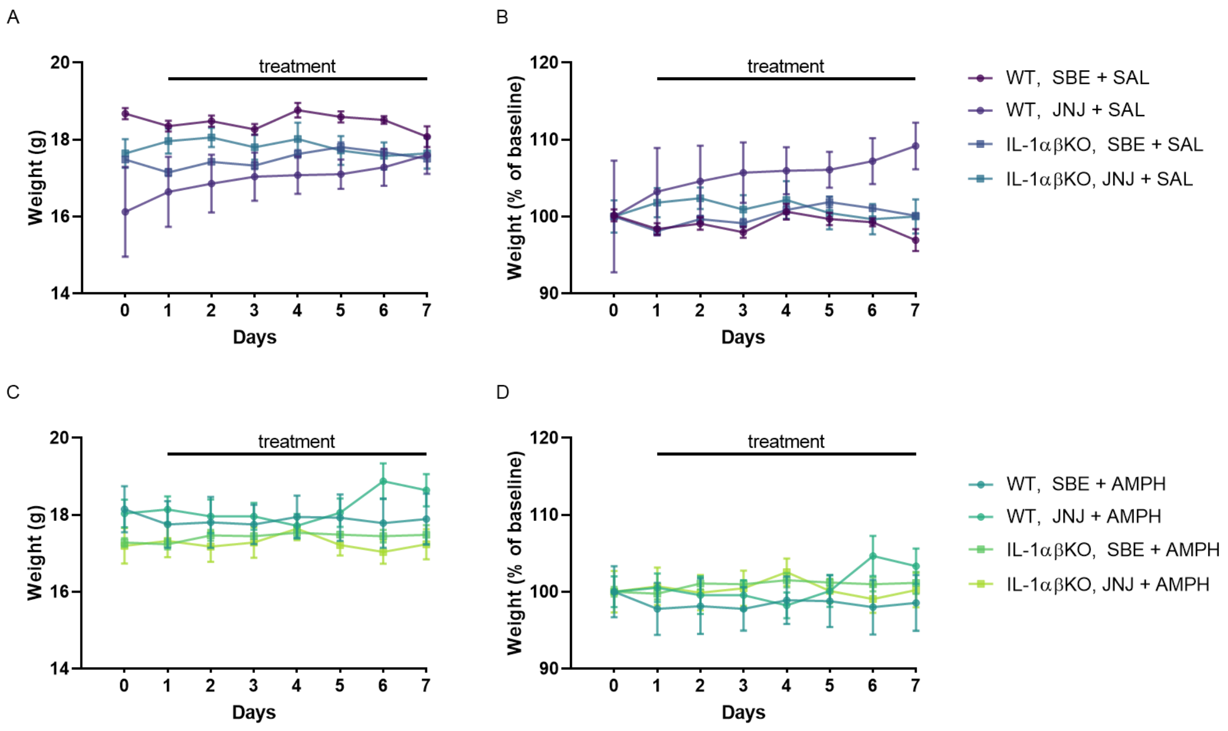

Figure S6. Treatments did not cause any changes in the body weight of female mice, related to Figure 11. (A, C) Mice were weighed daily for 8 days. (B, D) Weight % of baseline. Values are presented as means ± SEM of n=5-5 mice per group. Repeated Measures ANOVA (A, B, C, D) followed by Tukey's multiple comparison *post hoc* test. AMPH, d-amphetamine, ANA, anakinra, IL-1αβKO, interleukin-1α/β knockout mice, JNJ, JNJ-47965567, MCC, MCC 950, SAL, saline, SBE, beta cyclodextrin sulfobutyl ethers, WT, wild-type mice.

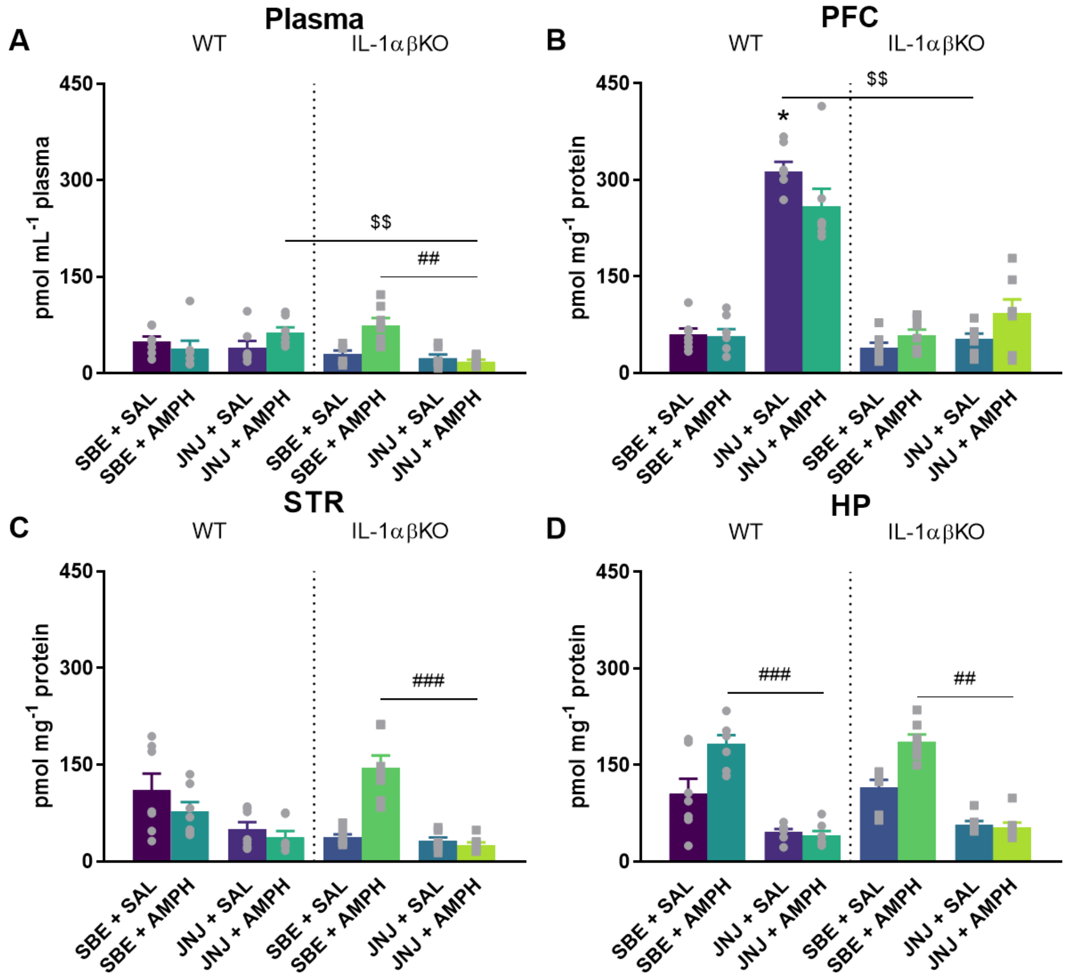

Figure S7. JNJ significantly decreased AMPH-induced 5-HT levels in plasma (A) and STR (C) in IL-1αβKO mice and HP (D) in both genotype, related to Figure 3. 5-HT levels were analysed using HPLC in the plasma, prefrontal cortex (PFC), striatum (STR) and hippocampus (HP). Values are presented as means ± SEM of n=7 mice per group. Kruskal-Wallis test with multiple comparisons: *p < 0.05 compared to SBE + SAL treated group in a same genotype; $^{\#}p < 0.05$, $^{\#\#}p < 0.01$, $^{\#\#\#}p < 0.001$ compared to SBE + AMPH treated group in a same genotype; $^{\$\$}p < 0.01$ compared to the same treatment group with WT. AMPH, d-amphetamine, IL-1αβKO, interleukin-1α/β knockout mice, JNJ, JNJ-47965567, SAL, saline, SBE, beta cyclodextrin sulfobutyl ethers, WT, wild-type mice, 5-HT, serotonin.

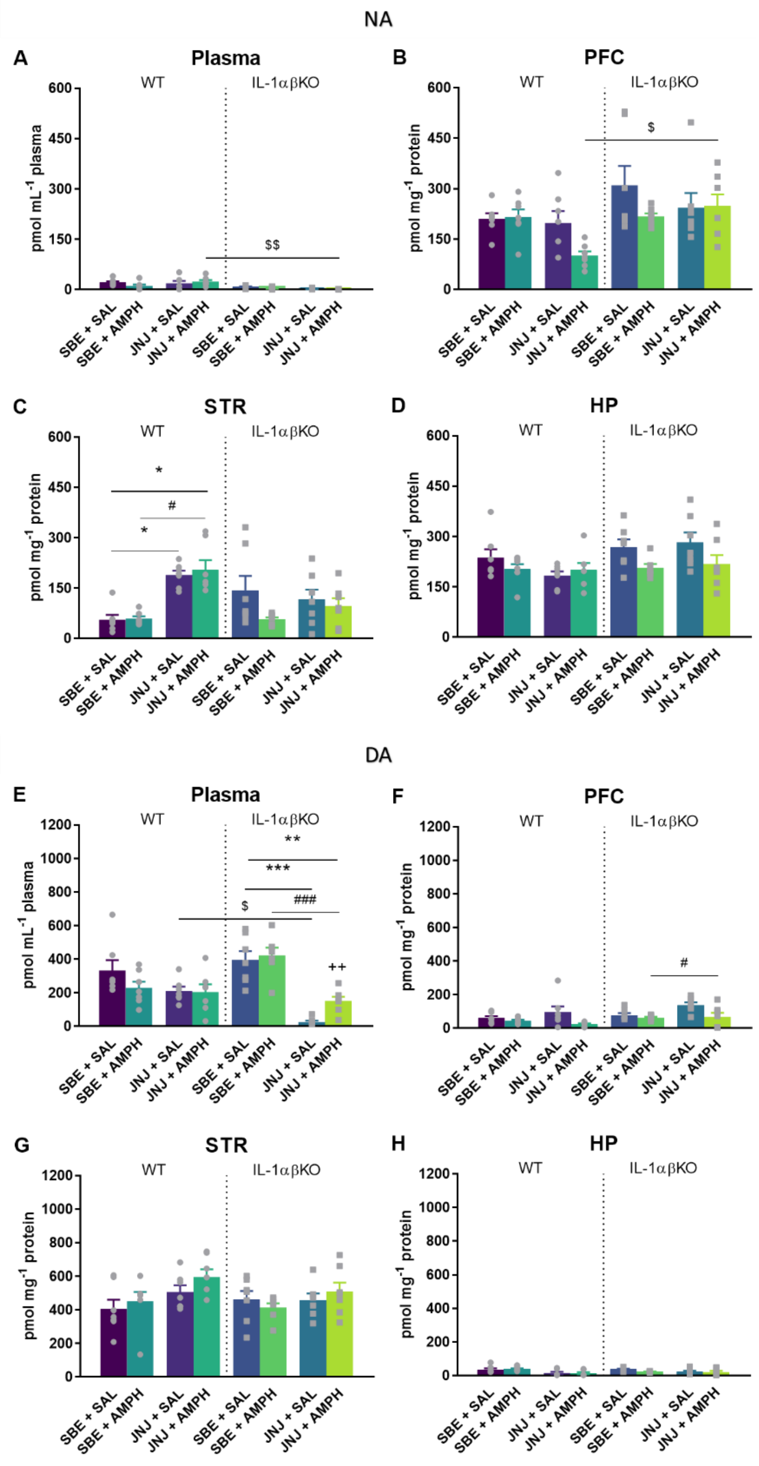

Figure S8. NA and DA levels in the plasma, PFC, STR and HP 1 hour after last JNJ / SBE + AMPH / SAL injection in WT and IL-1αβKO mice, related to Figure 3. NA and DA levels were analysed using HPLC in the plasma, prefrontal cortex (PFC), striatum (STR) and hippocampus (HP). Values are presented as means ± SEM of n=7 mice per group. Two-way ANOVA followed by Tukey's multiple comparison *post hoc* test (E and F (after square root transformation), G) or Kruskal-Wallis test (A, B, C, D, H): $^{*}p < 0.05$, $^{**}p < 0.01$, $^{***}p < 0.001$ compared to SBE + SAL treated group in a same genotype; $^{\#}p < 0.05$, $^{\#\#\#}p < 0.001$ compared to SBE + AMPH treated group in a same genotype; $^{++}p < 0.01$ compared to JNJ + SAL treated

group in a same genotype; $^{\$}p < 0.05$, $^{\$\$}p < 0.01$ compared to the same treatment group with WT. AMPH, d-amphetamine, DA, dopamine, HP, hippocampus, IL-1αβKO, interleukin-1α/β knockout mice, JNJ, JNJ-47965567, NA, noradrenaline, PFC, prefrontal cortex, SAL, saline, SBE, beta cyclodextrin sulfobutyl ethers, STR, striatum, WT, wild-type mice.

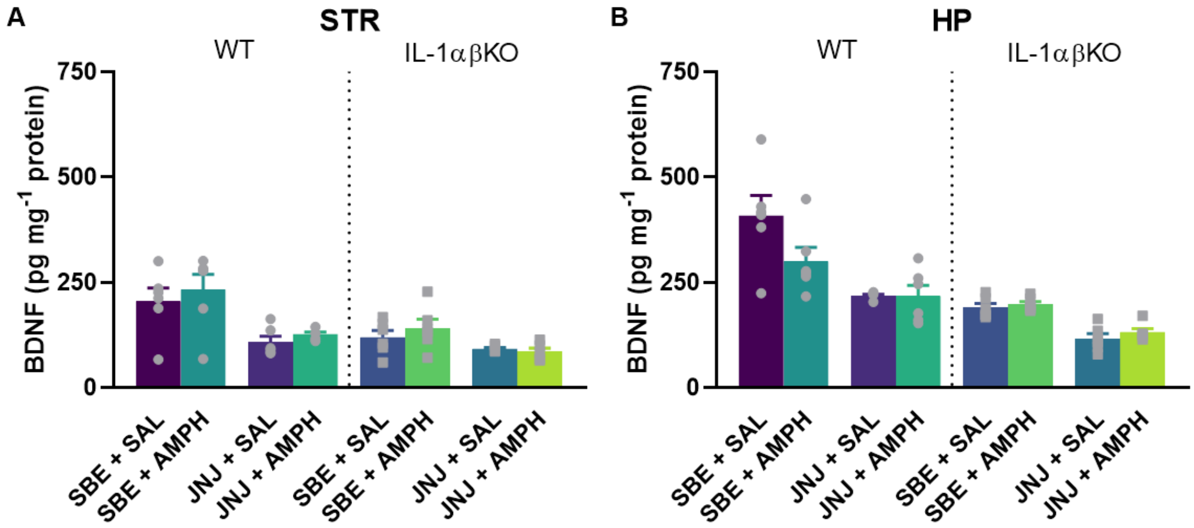

Figure S9. BDNF levels in the STR and HP 1 hour after last JNJ / SBE + AMPH / SAL injection in WT and IL-1αβKO mice, related to Figure 3. To evaluate the effect of repeated AMPH on the BDNF levels in STR and HP, we measured them by ELISA. Repeated administration of JNJ and AMPH did not affect striatal and hippocampal levels of BDNF in both WT and IL-1αβKO mice compared with the SBE + SAL group. Values are presented as means ± SEM of n=6 mice per group. Kruskal-Wallis test with multiple comparisons (A, B). AMPH, d-amphetamine, BDNF, brain-derived neurotrophic factor, HP, hippocampus, IL-1αβKO, interleukin-1α/β knockout mice, JNJ, JNJ-47965567, SAL, saline, SBE, beta cyclodextrin sulfobutyl ethers, STR, striatum, WT, wild-type mice.

References

1. Enman, N.M., and Unterwald, E.M. (2012). Inhibition of GSK3 attenuates amphetamine-induced hyperactivity and sensitization in the mouse. Behav Brain Res *231*, 217-225. 10.1016/j.bbr.2012.03.027.
2. Dal-Pont, G.C., Jorio, M.T.S., Resende, W.R., Gava, F.F., Aguiar-Geraldo, J.M., Possamai-Della, T., Peper-Nascimento, J., Quevedo, J., and Valvassori, S.S. (2019). Effects of lithium and valproate on behavioral parameters and neurotrophic factor levels in an animal model of mania induced by paradoxical sleep deprivation. J Psychiatr Res *119*, 76-83. 10.1016/j.jpsychires.2019.09.003.
3. Gubert, C., Andrejew, R., Leite, C.E., Moritz, C.E.J., Scholl, J., Figueiro, F., Kapczinski, F., da Silva Magalhaes, P.V., and Battastini, A.M.O. (2020). P2X7 Purinergic Receptor Is Involved in the Pathophysiology of Mania: a Preclinical Study. Mol Neurobiol *57*, 1347-1360. 10.1007/s12035-019-01817-0.
